# Iron‐Single‐Atom Nanozyme with NIR Enhanced Catalytic Activities for Facilitating MRSA‐Infected Wound Therapy

**DOI:** 10.1002/advs.202308684

**Published:** 2024-02-08

**Authors:** Qian Liu, Xueliang Liu, Xiaojun He, Danyan Wang, Chen Zheng, Lin Jin, Jianliang Shen

**Affiliations:** ^1^ National Engineering Research Center of Ophthalmology and Optometry Eye Hospital Wenzhou Medical University Wenzhou Zhejiang 325027 P. R. China; ^2^ Zhejiang Engineering Research Center for Tissue Repair Materials Wenzhou Institute University of Chinese Academy of Sciences Wenzhou Zhejiang 325001 P. R. China; ^3^ The Key Laboratory of Rare Earth Functional Materials and Applications International Joint Research Laboratory for Biomedical Nanomaterials of Henan Zhoukou Normal University Zhoukou 466001 P. R. China; ^4^ School of Pharmaceutical Sciences Wenzhou Medical University Wenzhou Zhejiang 325035 P. R. China; ^5^ College of Life and Environmental Science Wenzhou University Wenzhou Zhejiang 325035 P. R. China

**Keywords:** antibacterial therapy, iron, nanozyme, single‐atom catalyst, wound healing

## Abstract

Patients with methicillin‐resistant *Staphylococcus aureus* (MRSA) infections may have higher death rates than those with non‐drug‐resistant infections. Nanozymes offer a promising approach to eliminating bacteria by producing reactive oxygen species. However, most of the conventional nanozyme technologies encounter significant challenges with respect to size, composition, and a naturally low number of active sites. The present study synthesizes a iron‐single‐atom structure (Fe‐SAC) via nitrogen doped‐carbon, a Fe‐N_5_ catalyst (Fe‐SAC) with a high metal loading (4.3 wt.%). This catalyst permits the development of nanozymes consisting of single‐atom structures with active sites resembling enzymes, embedded within nanomaterials. Fe‐SAC displays peroxidase‐like activities upon exposure to H_2_O_2_. This structure facilitates the production of hydroxyl radicals, well‐known for their strong bactericidal effects. Furthermore, the photothermal properties augment the bactericidal efficacy of Fe‐SAC. The findings reveal that Fe‐SAC disrupts the bacterial cell membranes and the biofilms, contributing to their antibacterial effects. The bactericidal properties of Fe‐SAC are harnessed, which eradicates the MRSA infections in wounds and improves wound healing. Taken together, these findings suggest that single Fe atom nanozymes offer a novel perspective on the catalytic mechanism and design, holding immense potential as next‐generation nanozymes.

## Graphical abstract

A high‐metal‐loading Fe single‐atom catalyst with peroxidase‐like activity and good photothermal performance is designed for antibacterial effects and treating methicillin‐resistant *Staphylococcus aureus*‐infected wound therapy.

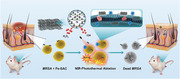

## 1 Introduction

Antimicrobial and drug resistance are major global public health concerns. The misuse of antibiotics has exacerbated these phenomena, making it crucial to develop alternative antibacterial agents.^[^ ^1^ ^]^ With the diminishing efficacy of antibiotics, several infections, such as pneumonia, tuberculosis, sepsis, gonorrhea, and foodborne illnesses, are becoming difficult to treat and even untreatable. Biofilms, complex communities of bacteria, that provide a protective barrier and enhance antibiotic resistance. In addition, bacteria in the biofilm could exhibit about 1000‐fold antibiotic resistance.^[^ ^2^ ^]^ The emergence of drug‐resistant bacteria has made otherwise common infections difficult to treat, increasing patient suffering and mortality.^[^ ^3^ ^]^ Presently, drug‐resistant strains have spread widely worldwide, including drug‐resistant *Streptococcus pneumoniae*, methicillin‐resistant *Staphylococcus aureus* (MRSA), and multidrug‐resistant *Tuberculosis*.^[^ ^1^ ^]^ These strains are resistant to conventional and last‐line antibiotics, further limiting the treatment options. Therefore, new approaches to resolve those issues are imperative.

Nanozyme is an enzyme molecule with nanoscale size, a high specific surface area, and specific catalytic performance and may have a potential usage in the antibacterial sector.^[^ ^4^ ^]^ Hitherto, the research on the antibacterial mechanism of reactive oxygen species (ROS) generated by nanozyme has mainly focused on two aspects. First is the generation of ROS through oxygen molecules, such as superoxide anion (O_2_ **·** ^−^), hydroxyl radical (**·**OH), and singlet oxygen (^1^O_2_), on the surface of nanozyme. ROS generation through catalytic processes primarily relies on the enzymatic activity of various enzymes. For example, nanozymes with peroxidase‐like properties, such as peroxidase (POD)‐mimic nanozymes, and convert hydrogen peroxide (H_2_O_2_) into highly cytotoxic hydroxyl radicals (**·**OH), thereby increasing intracellular oxidative stress. These robust ROS can eliminate bacterial cell membranes and DNA structures, exerting an antibacterial effect. The second is to generate ROS through transition metal ions on the surface of nanozyme. Such as copper ions (Cu^2+^) and silver ions (Ag^+^), these ions combine with bacterial proteins and DNA, destroying their normal functions, and thus achieving antibacterial effects.^[^ ^5^ ^]^ However, the antibacterial mechanism of nanozyme to generate ROS has some limitations. First, the antibacterial effect of nanozyme is affected by the stability and activity of the catalyst, and some catalysts may lose their activity after long‐term use, thereby reducing the antibacterial effect. Second, the antibacterial effect of nanozyme mainly focuses on the destruction of the outer membrane of the bacteria and DNA damage; thus it may be less effective for some bacteria and fungi with inner membranes and other microorganisms.^[^ ^5^ ^]^ Furthermore, due to anaerobic glycolysis, the unique microenvironment of infected areas is mildly acidic, hypoxic with overexpressed glutathione (GSH) than healthy tissues.^[^ ^6^ ^]^ This high GSH expression significantly inhibits the catalytic therapeutic effects of nanozymes.^[^ ^7^ ^]^ Hence, there is a pressing demand for antibacterial nanozymes that possess improved catalytic activity and ability to deplete GSH.

Single‐atom catalysts (SACs) that can maximize metal utilization constitute a novel enzyme simulation system with characteristics of metal single atoms and can exert enzyme‐like catalysis in catalytic reactions.^[^ ^8^ ^]^ Notably, Feng et al^[^ ^9^ ^]^ developed a spherical mesoporous nanozyme composed of single‐atom iron–nitrogen–carbon (Fe–N–C) that demonstrated enhanced catalytic performance (Km = 4.84 mmol L^−1^) and high photothermal conversion efficiency (23.3%). It is an appropriate approach for antibacterial therapy due to the synergistic effect of photothermal treatment and nanozyme catalysis, which can kill the bacteria. In a study by Xu et al.^[^ ^8c^ ^]^ a zinc‐based zeolitic‐imidazolate‐framework (ZIF‐8) was used as a precursor to construct carbon nanoparticles with a porphyrin‐like structure around a single zinc atom. These nanoparticles exhibited remarkable peroxidase‐like activity, surpassing that of pure Fe_2_O_3_ and nanocarbon materials. The improved catalytic performance of these nanoparticles was attributed to the active sites, composed of individual zinc atoms. Hou et al.^[^ ^10^ ^]^ synthesized nitrogen‐doped carbon single iron atoms nano‐catalysts (SAF NCs) that catalyzed the peroxidase‐like reaction (Km = 11.95 µM), generating hydroxyl radicals (•OH) in the presence of H_2_O_2_ at physiological concentrations to eradicate bacteria. Similarly, Huang et al.^[^ ^4c^ ^]^ created a single‐atom nanozyme with a carbon framework and a confined axial N‐coordinated single‐atom iron to replicate the axial ligand‐coordination structure of nitrogen atoms in natural heme.

When adjusted for metal concentration, these FeN_5_ SA/CNF nanozymes exhibited oxidase‐like activity 70 times higher than commercial Pt/C and have been effectively used in mice skin wound models as an antibacterial treatment. Despite these significant advancements, SACs face certain challenges. Its catalytic activity does not yet match that of real enzymes, which could be attributed, at least partially, to the low metal atom content, especially in reported SAC materials.

Interestingly, carbon‐based SACs possess remarkable properties, such as affordability, adjustable porosity, surface properties, high specific surface area, and excellent conductivity. Pyrolysis method is widely used for preparing SACs with isolated metal–nitrogen–carbon (M–N–C) active sites, where metal‐doped zeolitic imidazolate frameworks (ZIFs) have been employed to synthesize SACs of various metals (Fe, Cu, Co, Ni, and Pt).^[^ ^11^ ^]^ Nonetheless, the use of metal‐organic frameworks (MOFs) for SAC synthesis faces challenges such as low product yield and metal conversion efficiency, limiting scalability. Previous studies have focused on SACs with metal content <1.5 wt.% to prevent particle aggregation,^[^ ^12^ ^]^ but this low metal loading and inefficient metal salt conversion hinder practical applications of SACs. Recent studies have reported that the electrocatalytic performance of SACs is strongly influenced by their unique coordination structures (M‐Nx), with the oversaturated M‐N_5_ structure exhibiting superior catalytic activity compared to the commonly known M‐N_4_ structure.^[^ ^13^ ^]^ The atomically dispersed M‐N_5_ moieties embedded in a carbon matrix doped with nitrogen can decrease reaction activation energy and increase catalytic rate in chemical catalysis through a coordinated environment and optimized electronic structure.^[^ ^14^ ^]^ Despite their close resemblance to natural enzymes and potential for superior enzyme‐like activity, the controlled preparation of single‐atom nanozymes with five‐coordinated structures has received scant attention.^[^ ^15^ ^]^ However, due to the uncontrollable synthesis of supersaturated coordination SA catalysts, only a few studies have focused on introducing SA catalysts with supersaturated M‐N_5_ sites into catalytic reactions. Therefore, an environment‐friendly method for synthesizing SACs with high metal conversion and loading is essential.

Iron is a commonly used catalyst in various industrial processes, such as the Haber–Bosch method for ammonia synthesis.^[^ ^16^ ^]^ It is also a vital catalyst in various biological processes and is essential for maintaining the function of enzymes, proteins, and transcription factors involved in diverse wound‐healing processes.^[^ ^16^ ^]^ Bacteria are commonly detected in wounds, and neutrophils can undergo a process termed netting to provide additional protection for the bacteria, making treatment challenging. The Arrhenius equation suggests that an increase in temperature boosts the production of ROS.^[^ ^17^ ^]^ Hence, the integration of peroxidase catalytic activity with photothermal therapy (PTT) is being explored as a potential strategy to combat antibiotic resistance. PTT is a treatment for bacterial infections that are both minimally invasive and can be controlled. It uses photothermal reagents to generate local hyperthermia under near‐infrared irradiation (NIR), but excessive power can be detrimental to healthy tissues.^[^ ^18^ ^]^ Furthermore, the temperature of thermal ablative for eukaryotic cells is above 45 °C, while that for prokaryotic cells is above 65 °C.^[^ ^19^ ^]^ This phenomenon indicates that high temperatures can be hazardous to cells, especially while eradicating biofilms or targeting germs that are resistant to drugs. Combination therapies may include lower temperatures than single PTT, and the resulting hyperthermia can strengthen the immune system by increasing the permeability of pathogenic cell membranes and drawing in an abundant immune cell. This improves the therapeutic benefits by fortifying the immune system and hastening the absorption of antibacterial medications. Hence, incorporating the peroxidase with PTT and amplifying the production of catalytic ROS can reduce the potential harm caused by PTT.

Herein, we successfully synthesized a Fe single‐atom catalyst (Fe‐SAC) with exceptional peroxidase‐like activity using a controlled metal atom‐releasing strategy. This Fe‐N_5_ catalyst with a metal loading of 4.3 wt.% was used to construct single‐atom nanozymes with enzyme‐like active sites in nanomaterials. The nanocomposites involving N‐doped carbon (NC) and graphitic carbon nitride doped with Fe (Fe/g‐C_3_N_4_) were produced through the low‐temperature pyrolysis of a mixture containing polyvinylpyrrolidone (PVP), urea, and ferric chloride (FeCl_3_). The Fe atoms were then gradually liberated through the decomposition of Fe/g‐C_3_N_4_ at high temperatures and simultaneously captured by NC, transforming into Fe single‐atom sites. Fe‐SAC presents excellent photothermal stability when exposed to NIR, high peroxidase‐like performance to catalyze H_2_O_2_ into generating **·**OH to kill MRSA around the wounds, and GSH‐depletion activity to alter the microenvironment in infection areas. Together, the present study offers a viable approach for addressing deeply entrenched MRSA‐infected wounds through a combined therapeutic platform.

## 2 Results and Discussion

### 2.1 Synthesis and Characterization of Fe‐SAC

**Scheme 1:**
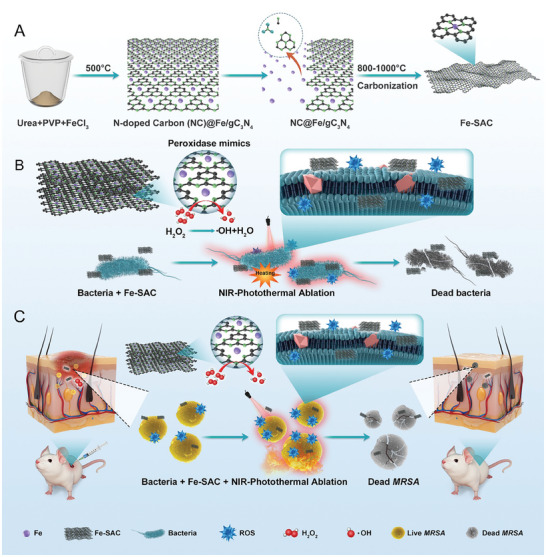
Fe‐SAC with peroxidase mimics activities and photothermal performance against bacteria. A) Synthetic process of Fe‐SAC. Illustration of Fe‐SAC against bacterial in vitro B) and in vivo C).

Fe‐SAC was prepared through a controllable metal atom releasing strategy (**Scheme** **1**). To prepare the Fe‐SAC catalyst, we pyrolyzed a mixture of polyvinylpyrrolidone (PVP), urea, and FeCl_3_·6H_2_O at low temperature, which resulted in nitrogen‐doped carbon (NC) and Fe‐doped graphitic carbon nitride (Fe/g‐C_3_N_4_) nanoparticles (NC@Fe/g‐ C_3_N_4_). Specifically, metal salts (FeCl_3_·6H_2_O) and urea undergo a gradual polymerization process, leading to the creation of a carbon nitride (Fe/g‐ C_3_N_4_) structure that is Fe‐doped and graphite‐like. Simultaneously, PVP is thermally decomposed, utilizing g‐ C_3_N_4_ as a template and thus producing flake NC nanomaterials that are coated upon the outside of Fe/g‐ C_3_N_4_. Upon surpassing a temperature of 600 °C, Fe/g‐ C_3_N_4_ decomposes gradually and releases doped Fe atoms in a controlled manner. These metal atoms are subsequently captured by the abundant nitrogen atom sites in the external NC material, thereby creating transition Fe single‐atom active sites (Fe‐N‐C). The controlled release of metal atoms is achieved through temperature adjustment and optimal heating rate. As the temperature continues to rise beyond 800 °C, g‐C_3_N_4_ decomposes entirely, resulting in the formation of a nitrogen‐doped carbon‐supported transition Fe single‐atom material (Fe‐SAC).

**Figure 1:**
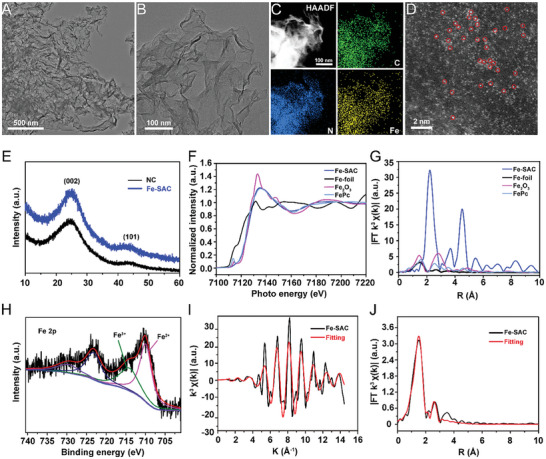
Characterization of the structure of Fe‐SAC. A) TEM images of synthetic Fe‐SAC. B) TEM images of synthetic Fe‐SAC with a scale bar of 100 nm. C) Corresponding AC‐STEM image and EDS elemental mapping (C, N, and Fe) of Fe‐SAC. D) High‐resolution HAADF‐STEM image of Fe‐SAC. The isolated single iron atoms against the amorphous substrate are shown by the circled red spots. E) XRD patterns of NC (black line) and synthetic Fe‐SAC (blue line). F) Fe K‐edge XANES spectra and G) Fourier‐transformed Fe K‐edge EXAFS spectra of Fe‐SAC, Fe foil, Fe_2_O_3,_ and FePC. H) High‐resolution XPS spectra of Fe 2p. I) EXAFS fitting outcome of Fe‐SAC in k space. J) EXAFS fitting result of Fe‐SAC in R space.

The morphology of Fe‐SAC was evaluated by transmission electron microscopy (TEM). The TEM images showed the sheet‐like structure of Fe‐SAC (**Figure** **1**A,B) and NC (Figure S1A,B, Supporting Information). The lack of metallic Fe or FeOx nanoparticles suggested a uniform distribution of Fe atoms. Figure 1C shows the mapping images from energy‐dispersive X‐ray spectroscopy (EDS), and the high‐angel annular dark‐field scanning TEM (HAADF‐STEM) demonstrated the consistent allocation of the elements carbon (C), nitrogen (N), and iron (Fe) in Fe‐SAC. Then, the dispersity of Fe atoms was confirmed by the aberration‐corrected scanning transmission electron microscope (AC‐STEM). Figure 1D shows a high density of bright white dots, indicating high metal loading and the presence of isolated Fe atoms in the Fe‐SAC catalyst. This phenomenon confirms the formation of single Fe atom active sites, without aggregation of iron nanoclusters or nanoparticles. The X‐ray diffraction (XRD) patterns of Fe‐SAC were similar to those of NC, as depicted in Figure 1E. Two broad diffraction peaks at 2θ of 25° and 43.8° matched the carbon atoms in the (002) and (101) planes.^[^ ^20^ ^]^ Strikingly, no characteristic diffraction peaks of Fe or FeOx nanoparticles appeared, which was consistent with the results of TEM. X‐ray photoelectron spectroscopy (XPS) was used to analyze the elemental composition and chemical environment of Fe‐SAC. In line with the results of the EDS mapping, the XPS survey indicated that the weight content of C, N, O, and Fe was computed to be 73.3%, 18.5%, 3.9%, and 4.3%, respectively. (Table S1, Supporting Information). The high level of N provided many active sites for anchoring Fe atoms. In the high‐resolution N 1s XPS spectra (Figure S2A, Supporting Information), the peak of binding energy at 398.9 eV confirmed the Fe‐N coordination.^[^ ^21^ ^]^ Also, four characteristic peaks corresponding to pyridinic N, pyrrolic N, graphitic N, and oxidized N could be fitted. The pyrrolic N and pyridinic N may act as attachment points for the Fe‐Nx moieties. Three peaks are observed in the C 1‐s spectra and were assigned to the C═C, C═N, and C─N bonds, respectively (Figure S2B, Supporting Information). Additionally, the XPS survey spectrum of Fe‐SAC showed the presence of C, O, and N (Figure S3, Supporting Information).

X‐ray absorption fine structure (XAFS) was used to investigate the coordination environment and chemical state of Fe atoms. Figure 1F shows the X‐ray absorption near‐edge structure (XANES) spectrum of Fe‐SAC compared to Fe foil, Fe_2_O_3,_ and iron phthalocyanine (FePC) references. The absorption energy position near the edge of Fe‐SAC fell between the standard Fe foil and Fe_2_O_3_, suggesting that the Fe atoms in Fe‐SAC were in an oxidation state ranging from +2 to +3. The Fourier transform (FT) k3‐weighted extended X‐ray absorption fine structure (EXAFS) spectra were displayed in Figure 1G. The two main peaks of Fe_2_O_3_ were at 1.46 and 2.80 Å, the two distinct peaks of Fe foil were at 2.22 and 4.50 Å, and the major peak of Fe‐SAC was detected at 1.52 Å. These findings substantiate the distribution of Fe atoms in the N‐doped carbon support. In Fe2p XPS spectra, a sharp peak at 710 and 714.3 eV attributed to Fe^2+^ 2p3/2 and Fe^3+^ 2p3/2 was observed for the Fe‐SAC (Figure 1H). The absence of a peak at ≈707 eV confirmed the absence of any metallic Fe species. Figure 1I,J depicts the outcomes of an EXAFS curve‐fitting analysis in k and R spaces to determine the coordination configure ratio of Fe. The fitting curves for EXAFS (Figure 1J) of Fe‐SAC revealed that the first shell coordination number of Fe atoms was 5.1, with Fe‐N_5_ activity sites, and the surrounding coordination atoms (N) and Fe atoms had a bond length of 2.01. The concentration of an isolated single iron atomic species was 4.3 wt.%, as calculated by inductively coupled plasma optical emission spectrometry (ICP‐OES). This finding is indicative of a high catalytic activity. Thus, a Fe single‐atom catalyst was successfully prepared with Fe‐N_5_ active sites and metal loading of 4.3 wt.%.

### 2.2 NIR‐I Irradiation Enhances Peroxidase‐Like Activity of Fe‐SAC

**Figure 2:**
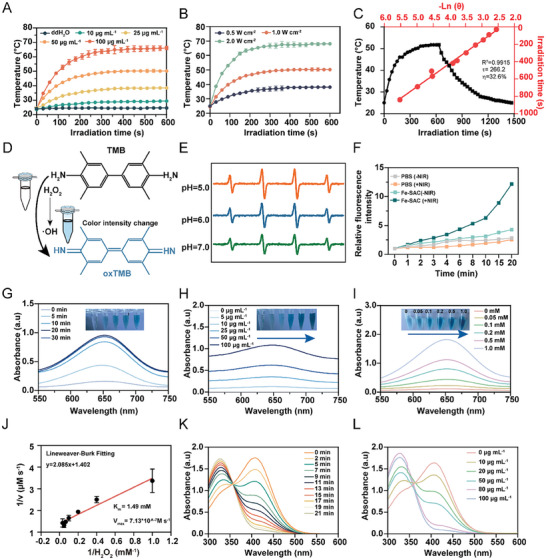
NIR‐I enhances peroxidase‐like activity of Fe‐SAC. A) Temperature variation profiles of various concentrations of Fe‐SAC post‐exposure to an 808 nm laser (1.0 W cm^−2^, 10 min). B) Temperature progression of a dispersed Fe‐SAC suspension at 50 µg mL^−1^ under various NIR irradiation intensities. C) Temperature change during a single heating and cooling cycle and the linear regression curve of ‐Ln(θ) versus Fe‐SAC during the cooling phase. D) Schematic illustration depicting the peroxidase‐like behavior of Fe‐SAC through the TMB colorimetric mechanism. E) ESR spectrum of hydroxyl radical generated from the Fenton reaction and trapped by DMPO under different pH conditions. F) Relative fluorescence changes of DCFH‐DA without or with NIR‐I irradiation. Time G), concentration H), and concentration of H_2_O_2_‐dependent I) absorption spectra of a dispersed Fe‐SAC suspension. Digital images display differences in the color of related samples. J) Michaelis‐Menten kinetic analysis of Fe‐SAC with H_2_O_2_ as a substrate. Time‐dependent K) and concentration‐dependent L) absorption spectra of consumed GSH after treatment with Fe‐SAC suspension.

NIR absorption is the basic characteristic of a good photothermal agent. The near‐infrared light absorption characteristics of Fe‐SAC were assessed using an UV–vis–NIR spectrometer. Upon exposure to UV–vis–NIR light, Fe‐SAC displays extensive absorption of NIR‐I and NIR‐II energy lasers (Figure S4A, Supporting Information). The UV absorption spectrum of Fe‐SAC has a significant absorption peak at 800–950 nm compared to NC (Figure S4B, Supporting Information). Notably, the degree of absorption exhibited by Fe‐SAC in the NIR‐I region surpassed that of NC which lacked Fe doping, as illustrated in Figure S5 (Supporting Information). A metal material exhibiting NIR‐responsive properties induces local hyperthermia upon exposure to NIR light, rendering it a promising candidate for initiating PTT to eradicate bacteria. The photothermal characteristics of Fe‐SAC dispersed solution (0, 10, 25, 50, and 100 µg mL^−1^) were subjected to NIR‐I (808 nm, 1.0 W cm^−2^) for 10 min. According to **Figure** **2**A, the temperature variations of Fe‐SAC dispersion were positively correlated with the concentration of the nanoparticles. Also, the temperature reached 40.0, 50.2, and 65.4 °C within 5 min at 25–100 µg mL^−1^, whereas the temperature of water barely reached 28 °C.

Conversely, when the power density of the NIR light increased to 2.0 W cm^−2^, the temperature of 50 µg mL^−1^ Fe‐SAC solution rose from 36.5 to 67.9 °C (Figure 2B). The real‐time temperature, measured by an infrared thermal imaging camera (TIC), was presented in Figure S6, (Supporting Information). Constant healing and cooling are employed for photothermal stability as it is essential for PTT. Figure S7 (Supporting Information) shows negligible temperature changes in a recycling heating and cooling procedure over five on/off cycles, further forming the excellent photothermal ability of Fe‐SAC. Based on the healing‐cooling curve and corresponding thermal time constant (*τ_s_*),^[^ ^22^ ^]^ the preeminent photothermal conversion efficiency (*η*) of Fe‐SAC was determined as 32.6% (Figure 2C), which could be explained by its intense absorption in NIR.

The emergence of nano‐catalytic drugs offers new possibilities for antibacterial therapy. 3,3′,5,5′‐tetramethylbenzidine (TMB) is a sign of •OH, a catalytic reaction that catalyzes an oxidation reaction between reactive oxygen species and TMB.^[^ ^23^ ^]^ The peroxidase‐like catalytic activity of Fe‐SAC was measured by TMB and H_2_O_2_ (Figure 2D); no changes were observed in color when deionized water was used alone (Figures S8 and S9, Supporting Information). Similarly, the NC did not exhibit any color change under different pH conditions. However, in the presence of Fe‐SAC and H_2_O_2_, a distinct peak emerged at 652 nm in the TMB signal (Figure S10, Supporting Information). Also, only Fe‐SAC dispersion could oxidase TMB and obtain absorption at 652 nm. Together, these findings demonstrated that Fe‐SAC oxidizes TMB to form oxTMB; this reaction is further strengthened by H_2_O_2_ addition. Subsequently, the presence of •OH was verified via electron spin resonance (ESR) spectroscopy^[^ ^24^ ^]^ and using 5‐tert‐butoxycarbonyl‐5‐pyrroline N‐oxide (BMPO) as a ROS scavenger^[^ ^24^ ^]^ (Figure 2E). The addition of Fe‐SAC and a physiological amount of H_2_O_2_ (100 × 10^−6^ M) facilitates efficient absorption of the released hazardous •OH by BMPO, leading to the development of identifiable •OH/BMPO adducts with distinct signals of 1:2:2:1 in a neutral buffer solution (pH 7.0). This indicates the catalyst's ability to generate radicals. Interestingly, the signal's spectrum of the collected radical adducts increased noticeably when the system was co‐incubated in an acidic buffer (pH 5.0 or 6.0) to that in the neutral buffers. This acidity‐responsive •OH generation shows a marked specificity toward mildly acidic pathological abnormalities. Additionally, Fe‐SAC could generate other radicals, such as ^1^O_2_ and O_2_·^−^ (Figures S13 and S14, Supporting Information). A cell‐permeable probe called DCFH‐DA is utilized to detect ROS. After irradiation by NIR‐I (808 nm, 5 min), Fe‐SAC generates abundant ROS and enhances the catalytic activity (Figure 2F). In the event of prolonged reaction (Figure 2G) or increased concentration of Fe‐SAC 0 to 100 µg mL^−1^ (Figure 2H), more oxTMB absorption was observed at 652 nm. Also, higher H_2_O_2_ concentrations enhance the absorption of oxTMB at 652 nm., indicating the peroxidase‐like catalytic performance of Fe‐SAC (Figure 2I). Nanozyme as a kind of catalyst, pH, and temperature also control its catalytic activity.^[^ ^22^ ^]^ Different ratios of phosphate and sodium citrate in the buffer revealed that the pH range between 4.0 and 6.0 guarantees a high peroxidase‐like catalytic performance of Fe‐SAC (Figure S11, Supporting Information). However, NC did not oxidase TMB at any pH condition (Figure S9, Supporting Information). Due to its high maximum reaction velocity (Vmax) and low Michaelis–Menten constants (Km), the catalytic performance indicated an improved interaction between the catalyst and the substrate. According to the Lineweaver‐Burk plot (Figures S14 and S15, Supporting Information), the Fe‐SAC displayed a Vmax of 4.05 × 10^−7^ M s^−1^ and a Km of 4.73 mM when H_2_O_2_ was applied as the substrate at ambient temperature (26 °C). Interestingly, under NIR‐I illumination (50°C), the Vmax and Km values were elevated to 7.13 × 10^−7^ M s^−1^ (1.49 mM), respectively (Figure 2J). This phenomenon indicated that temperature elevation boosts the peroxidase‐like performance of Fe‐SAC under laser (808 nm, 1.0 W cm^−2^) illumination. Importantly, Fe‐SAC exhibits remarkable catalytic activity, surpassing that of other advanced single‐atom nanozymes (Table S2, Supporting Information), affirming a heightened affinity of Fe‐SAC toward H_2_O_2_, ultimately augmenting its peroxidase activity for efficient function as a nanozyme.

Glutathione (GSH) is an important antioxidant that removes free radicals under physiological conditions, protecting the sulfhydryl groups in the proteins and enzymes. It also counteracts the increased concentration of •OH generated by the peroxidase‐like activity of Fe‐SAC; however, the concentration rises significantly at the infection sites owing to abnormal extracellular polymeric substance encapsulation.^[^ ^7a^ ^]^ The characteristic peaks of DTNB at 412 nm were decreased significantly in a time (Figure 2K) and concentration‐dependent manner (Figure 2L) within 21 minutes post‐treatment with Fe‐SAC. The increased concentration of •OH generated by the peroxidase‐like activity due to GSH depletion was evaluated using 5, 5′‐dithiobis (2‐nitrobenzoic acid) (DTNB). Also, Fe‐SAC showed Michaelis‐Menton kinetics during the catalytic reaction of GSH (Figure S16, Supporting Information). A high Km and Vmax were calculated to be 0.24 mM and 5.64 × 10^−6^ M s^−1^, respectively, manifesting the remarkable GSH‐depletion performance with high substrate affinity (Figure S17, Supporting Information).

### 2.3 Antibacterial Performance of Fe‐SAC In Vitro

**Figure 3:**
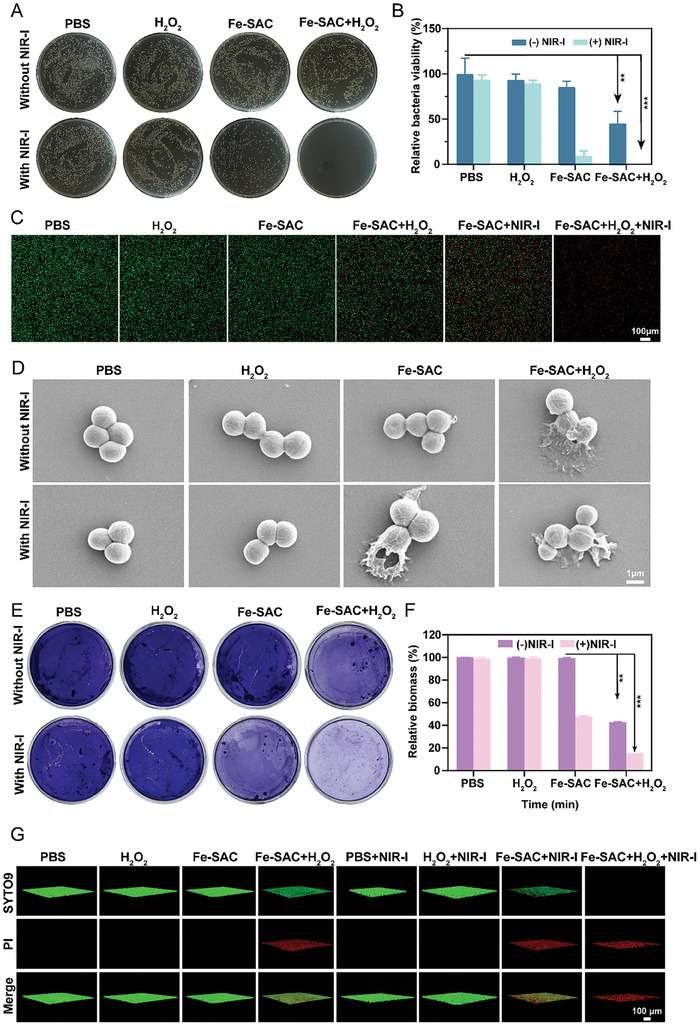
Antibacterial activities of Fe‐SAC in vitro. A) Agar plate photographs of MRSA bacterial colonies by Fe‐SAC under NIR‐I irradiation; PBS was as a control. B) Relative bacterial viability of MRSA. C) Live/dead staining of MRSA. D) SEM images of MRSA following various therapies. E) Images of MRSA biofilms, with PBS as a control, and F) corresponding quantitation of the biofilm biomass. G) 3D images of MRSA biofilm using confocal scanning microscopy. Data from each of the three independent experiments (n = 3) are presented as mean ± standard deviation (SD). The following criteria were used to assess statistical significance: ^*^ *p* < 0.05, ^**^ *p* < 0.01, ^***^ *p* < 0. 001.

Based on the peroxidase‐like performance of Fe‐SAC, we further explored the antibacterial effect against MRSA and *Escherichia coli* (*E*. *coli*). The antibacterial activity was assessed by colony formation assay. Also, to ensure the safety of organisms, the potential toxicity of large amounts of Fe‐SAC and H_2_O_2_ was investigated. The bacterial suspensions were co‐cultured with varying concentrations of Fe‐SAC and H_2_O_2_. The results showed that Fe‐SAC (0–200 µg mL^−1^) and H_2_O_2_ (0.1 mM) had negligible bactericidal effects on MRSA (Figure S18–S19, Supporting Information). Owing to the former photothermal effect in Figure 2A,B, we selected 50 µg mL^−1^ of Fe‐SAC and 0.1 mM H_2_O_2_ for subsequent experiments. Eight groups; 1) bacteria; 2) bacteria + H_2_O_2_; 3) bacteria + Fe‐SAC; 4) bacteria + Fe‐SAC + H_2_O_2_; 5) bacteria + NIR‐I light; 6) bacteria + NIR‐I light + H_2_O_2_; 7) bacteria + Fe‐SAC + NIR‐I light; 8) bacteria + Fe‐SAC + H_2_O_2_+ NIR‐I light, were applied to antibacterial performance of Fe‐SAC. **Figure** **3**A,B shows that Fe‐SAC alone has a slight antibacterial effect on Gram‐negative bacteria (A high Km and Vmax were calculated91.31%) as well as Gram‐positive bacteria (≈85.58%). In group four (bacteria + Fe‐SAC + H_2_O_2_), the relative bacterial viability of MRSA and *E. coli* dropped to 45.16% and 51.85%, respectively, suggesting that Fe‐SAC decomposes H_2_O_2_ to generate •OH to combat the microbes. In addition, under NIR‐I irradiation for 5 min, Fe‐SAC decreased the survival ratios of MRSA and *E. coli* to 9.39% and 31.17%, respectively. These data indicate that Fe‐SAC possesses a broad spectrum of bacterial activities, and cannot eliminate bacteria through catalytic or photothermal characteristics. Furthermore, in group 8 (bacteria + Fe‐SAC + H_2_O_2_+ NIR‐I light), the relative viability of MRSA and *E. coli* reached 99.8% and 98.5%, respectively, with photothermal plus chemotherapy (Figure 3A,B, Figure S20A,B, Supporting Information). Upon exposure to NIR‐I, its peroxidase‐like activity could be strengthened to eradicate bacterial infection. Additionally, live/dead staining (SYTO‐9 and PI) of the bacteria (Figure 3C, Figure S20C, Supporting Information) confirmed that the peroxidase‐like activity of Fe‐SAC is enhanced under NIR‐I irradiation. To elucidate the antibacterial process of Fe‐SAC, the morphological changes of MRSA and *E. coli* were explored further. Fe‐SAC + H_2_O_2_ mainly causes the surface collapse of bacteria (Figure 3D, Figure S20D, Supporting Information), and NIR decomposes bacterial cytoskeleton, leading to bacterial lysis and death.

Bacterial biofilm is a complex 3D structure formed by a community of bacteria and consists, composed of bacterial cells and adherent substances from the surrounding environment. Biofilms can attach to various surfaces, including both biological and non‐biological surfaces. They consist of multilayered cell aggregates, the extracellular matrix, and hydrated substances, which are stabilized by interactions between various molecules in the extracellular matrix. Bacterial biofilms protect against bacterial growth, reproduction, and survival, and exhibit high resistance to antibiotic treatment. Crystal violet staining assessed the impact of Fe‐SAC and H_2_O_2_ treatment on MRSA biofilms. A dark purple color indicated a high residual biofilm sedimentation. As shown in Figure 3E,F, Fe‐SAC, and H_2_O_2_ alleviate the formation of MRSA biofilms. The NIR‐I irradiation‐mediated biofilm disruption ratio was about 87.1%. The live/dead assay further investigated the cell membrane integrity by confocal microscopy. Living bacteria labeled with SYTO‐9 were represented by green fluorescence, whereas dead bacteria labeled with PI were indicated by red fluorescence. The Fe‐SAC + H_2_O_2_ + NIR‐I group showed more red fluorescence than the PBS group (Figure 3G), suggesting that this anti‐biofilm strategy could effectively eliminate biofilms.

### 2.4 Antibacterial Performance of Fe‐SAC In Vivo and Wound Healing

**Figure 4:**
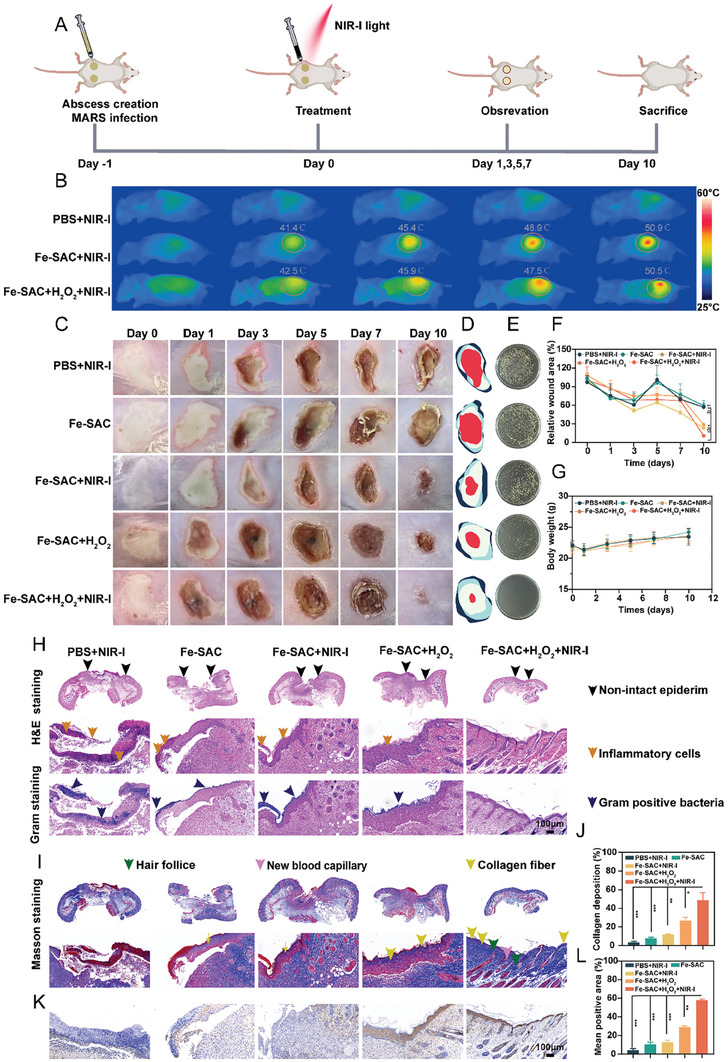
Fe‐SAC promoted infected wound healing and regeneration in vivo. A) Schematic of the experimental procedure for wound infection and treatment. B) Mice in the thermal pictures after treatment by PBS or Fe‐SAC suspensions (50 µg mL^−1^) and subjected to NIR‐I irradiation for 5 min. C) Photographs of MRSA‐infected wounds. D) Representation of wound healing trace in real‐time and E) corresponding pictures of the MRSA colonies for every group under treatment were obtained on the 10^th^ day of the assay. F) Quantitative statistics of the relative wound size in a time‐dependent manner. G) The mice's body weight was recorded while they were receiving treatment. H) Histologic changes were examined using H&E staining and Gram staining. I) Masson's staining and J) quantitative data of collagen deposition of various therapies. K) Immunohistochemical staining of VEGF and L) mean positive area analyst of VEGF. Data from each of the three independent experiments (n = 3) are presented as mean ± standard deviation (SD). The following criteria were used to assess statistical significance: ^*^ *p* < 0.05, ^**^ *p* < 0.01, ^***^ *p* < 0.001.

Owing to its excellent antibacterial properties and activity of eluding MRSA biofilm formations in vitro, we established an MRSA‐infected sub‐cutaneous abscess model for subsequent research into Fe‐SAC (**Figure** **4**A). Balb/c mice were split into five groups randomly (every group: n = 5): (1) PBS + NIR‐I, (2) Fe‐SAC, (3) Fe‐SAC + NIR‐I, (4) Fe‐SAC + H_2_O_2_, (5) Fe‐SAC + H_2_O_2_+NIR‐I. The retention and target of Fe‐SAC at the infected site were observed using a thermal imaging infrared camera. In Figure 4B, compared to PBS, Fe‐SAC treated groups could monitor temperature changes; the temperature reached 50.5‐50.9 °C in 5 min post‐irradiation by NIR‐I (the red spot in a yellow circle). This indicated that Fe‐SAC enriched in the infected area, providing a local hyperthermal activity to kill bacteria in vivo.

After different treatments, the condition of infected areas was recorded by a digital camera, and the body weight of the mice was measured every two days. Typically, no mouse in either of the groups appeared to be severely malnourished, and preliminary findings indicated that there was no harm to the mice affected mice in the various treatment groups. Figure 4C displays each group's wound healing. On days 0, 1, and 3 post‐infection, the skin tissue of PBS + NIR‐I presented apparent abscess redness and swelling of the surrounding skin tissue. Interestingly, Fe‐SAC alleviates the above conditions. On day 10, the infected area was scabbed over, and the wound did not heal completely; this delayed healing could be due to the eschar over the wound in the PBS + NIR‐I groups (Figure 4C,D). On day 3, a discernible scab was formed in the wound region, suggesting a decline in the population of bacteria and a quick healing reaction in the group treated with Fe‐SAC + H_2_O_2_ + NIR‐I, showing a decrease in the number of germs and a rapid healing response. On day 10, the coating and homogeneity of diseased tissue in the wound area (Figure 4E) showed the combined antimicrobial efficacy of various groups against MRSA at the wound site. Compared to other groups, bacterial growth was minimal on the plate covered with a homogenate of infected tissue from mice given Fe‐SAC + H_2_O_2_ + NIR‐I, indicating strong antibacterial effects. Furthermore, the wound size in the Fe‐SAC + H_2_O_2_ + NIR‐I group was significantly reduced, only 10.78% compared to 65.17%, 59.40%, 23.3%, and 28.80% in the PBS + NIR‐I, Fe‐SAC, Fe‐SAC + NIR‐I, and Fe‐SAC + H_2_O_2_ groups, respectively (Figure 4F).

After treatment, the mice were euthanized, and their wounded skin was extracted for hematoxylin‐eosin staining (H&E) and Masson staining to assess the progress of wound healing. The infected tissues in the PBS treatment group exhibited significant keratinocyte thickening, characteristics of crusting tissues, and severe damage to skin structures compared to the intact skin tissue of normal mice (Figure 4H top and center). The infected tissues treated with Fe‐SAC and Fe‐SAC + NIR‐I still exhibited a thickened epidermis and many inflammatory cells. Conversely, the tissues treated with Fe‐SAC + H_2_O_2_ and Fe‐SAC + H_2_O_2_ + NIR‐I showed relatively complete skin structures but with incomplete repair, and only a few neutrophils were detected. However, the infected tissue treated with Fe‐SAC + H_2_O_2_ + NIR‐I displayed increased granulation tissue thickness and complete restoration of the dermal structure, resembling normal mice, along with minimal neutrophils, fresh hair follicles, and blood vessels, and successful scar tissue healing. These findings indicated that when exposed to NIR‐I irradiation, Fe‐SAC + H_2_O_2_ + NIR‐I had the most significant therapeutic effect on infected wounds in MRSA‐infected mice. Gram staining also indicated that there were almost no bacteria after the wound had fully healed by Fe‐SAC + H_2_O_2_ + NIR‐I (Figure 4H bottom). Later, during the healing process, fibroblasts and collagen were found to be essential in the contraction of the wound and scarring through cross‐linking. Masson staining revealed that Fe‐SAC + H_2_O_2_ + NIR‐I‐treated wounds exhibited massive collagen deposition, indicating successful recovery and maturation of the damaged tissues, closely resembling the healthy skin tissue (*p* < 0.01) (Figure 4I,J). Furthermore, the treated group exhibited an extensive region of newly formed collagen fibers, which were both continuous and densely packed, indicating a more robust ability to repair wounds compared to the other treatment groups. The newly formed tissue must be vascularized for wounds to heal effectively.

VEGF‐A, also known as VEGF (vascular endothelial growth factor A), is a crucial molecule in promoting blood vessel formation under the skin. It is also involved in the regulation of hair growth as well as the progression of skin conditions, such as psoriasis and skin cancer.^[^ ^25^ ^]^ During the proliferative phase, VEGF levels affect angiogenesis, granulation tissue production, wound strength, and the pace of wound closure/re‐epithelialization. Moreover, VEGF can encourage scar tissue formation during the scar formation/remodeling phase. Fe‐SAC + H_2_O_2_ + NIR‐I‐treated wounds presented a large number of VEGF‐positive cells by immunohistochemical (IHC) staining (Figure 4K). In addition, the relative fluorescence intensity of VEGF was 4.14%, 10.52%, 12.72%, 29.11%, and 58.07% for PBS + NIR‐I, Fe‐SAC, Fe‐SAC + NIR‐I, and Fe‐SAC + H_2_O_2_, respectively (Figure 4L). These results demonstrated that Fe‐SAC stimulates wound reconstruction and blood vessel formation.

**Figure 5:**
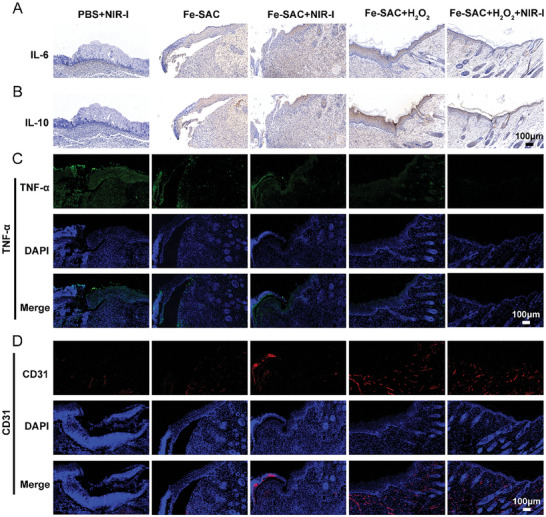
Immune microenvironment evaluation by immunohistochemical staining and immunofluorescence staining of wound area after various treatments. A) Immunohistochemical staining of IL‐6 (pro‐inflammatory factor) and IL‐10 B) (anti‐inflammatory factor). C) Immunofluorescence staining of TNF‐αand D) CD31 of infected wound areas by different treatments on the 10^th^ day.

Interleukin‐6 (IL‐6), classified as a cytokine, is a member of the chemokine family. In the event of inflammation or tissue damage, IL‐6 primarily controls and enhances immune responses triggers the synthesis of acute‐phase reactants and raises concentration.^[^ ^26^ ^]^ Conversely, interleukin‐10 (IL‐10) is a known immune‐suppressive molecule with inflammatory characteristics. It inhibits the release of inflammatory factors, participates in inflammatory and immunological reactions, and plays a crucial role in reducing inflammatory reactions and counteracting inflammatory mediators.^[^ ^24^, ^26^, ^27^ ^]^ As a mononuclear factor, tumor necrosis factor‐alpha (TNF‐α) can stimulate T lymphocytes to generate more inflammatory factors, which might trigger the inflammatory responses.^[^ ^28^ ^]^ TNF‐α immunofluorescence staining and IHC staining for IL‐6 and IL‐10 evaluated the degree of localized tissue inflammation in contaminated areas. As shown in **Figure** **5**A–C, skin tissue treated with PBS + NIR‐I exhibited a significant presence of densely packed neutrophils, confirming a severe inflammatory reaction, while the Fe‐SAC + NIR‐I, Fe‐SAC + H_2_O_2_, and Fe‐SAC + H_2_O_2_ + NIR‐I groups displayed a lower count of inflammatory cells, indicating the persistence of an inflammatory reaction. Also, a marked decrease in the levels of TNF‐α and IL‐6 in skin tissue following treatment with Fe‐SAC + H_2_O_2_ + NIR‐I treatment was noted. These results imply that Fe‐SAC + H_2_O_2_ + NIR‐I can decline both the inflammatory response in the infected tissue environment and repair the infected wounds. The histological analysis of angiogenesis involved immunofluorescence staining of CD31, a marker of vascular endothelial cells.^[^ ^29^ ^]^ The observed increase in the area positive for CD31 represents the vascular structure significantly in the Fe‐SAC + H_2_O_2_ + NIR‐I groups (Figure 5D), while Fe‐SAC + NIR‐I and Fe‐SAC + H_2_O_2_ treatments also demonstrated positive effects in expanding the region the CD31^+^, however, their results were found to be inferior to those of Fe‐SAC + H_2_O_2_ + NIR‐I treatment. Together, these findings demonstrated that Fe‐SAC therapy was effective in re‐establishing both functional and structural blood vessel networks in the wounds.

### 2.5 Biocompatibility Evolution of Fe‐SAC

**Figure 6:**
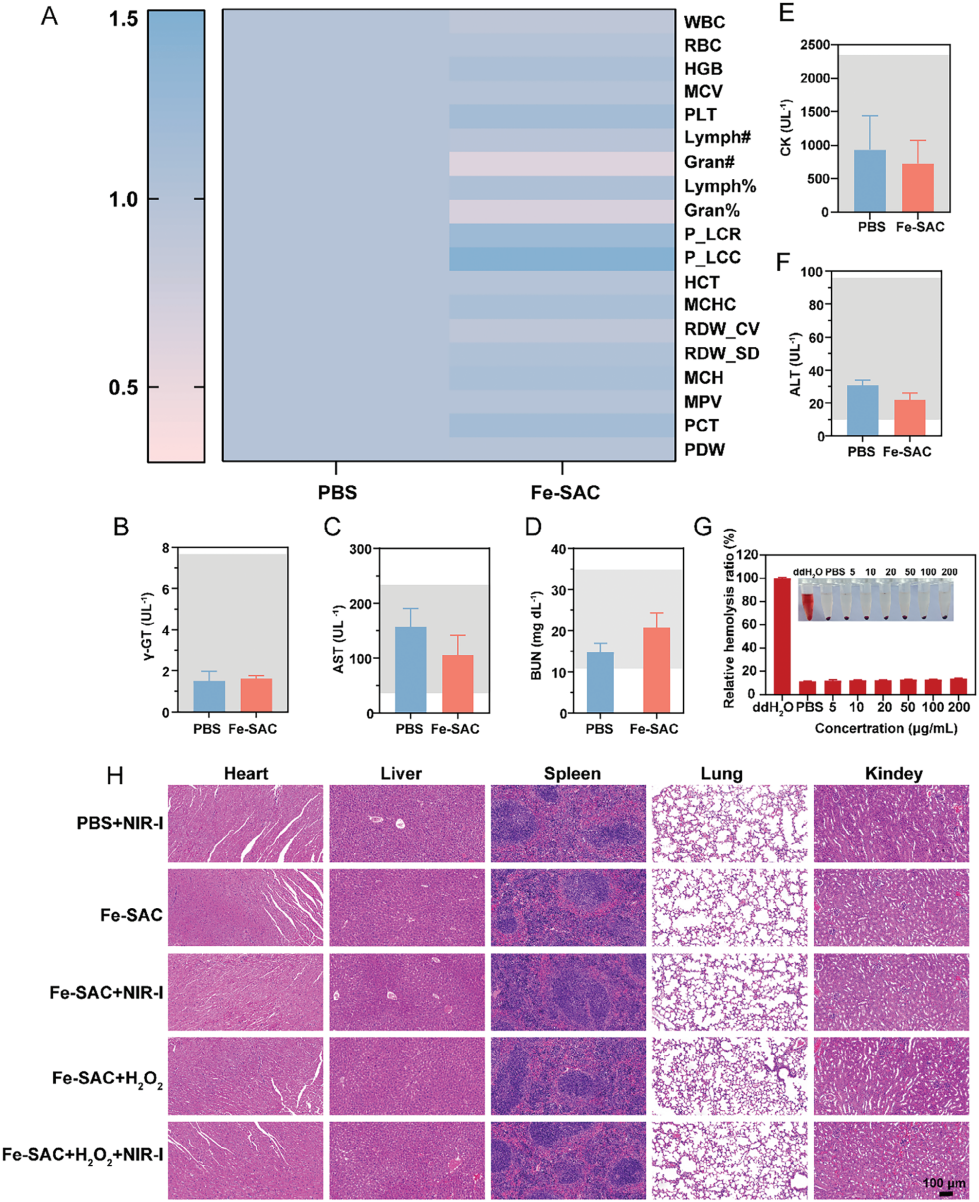
Biocompatibility of Fe‐SAC. A) Blood routine analysis of mice treated with Fe‐SAC after 48 h, healthy mice served as control. B– F) Blood serum biochemistry analysis of γ‐GT (B), AST (C), BUN (D), CK (E), ALT (F); blue indicates healthy mice, and red means Fe‐SAC treated mice. G) Relative hemolysis ratio of mouse RBSs after incubation with PBS, different concentrations of Fe‐SAC, and water at 37 °C for 3 h. H) The vital organs were subjected to H&E staining on the 10^th^ day following various treatments. Data from each of the three independent experiments (n = 3) are presented as mean ± standard deviation (SD).

To ensure the practical application of antibacterial agents, antibacterial effectiveness with minimal toxicity and excellent safety is vital. To assess the biosafety of Fe‐SAC, several tests, including cell toxicity assay, hemolysis assay, blood analysis, and in vivo toxicity examination, were conducted. For the cell toxicity assay, the effects of Fe‐SAC on L929 cells were determined. The results showed less cytotoxicity on L929 cells (Figure S21, Supporting Information). Similarly, the hemolysis assay, which measures the ability of Fe‐SAC to damage red blood cells, revealed no notable toxicity. For blood analysis, mice were injected with Fe‐SAC via intraperitoneal injection. After 48 h, the mice's physiological condition was assessed. Blood routine analysis (**Figure** **6**A) did not show remarkable abnormalities, indicating that Fe‐SAC did not have any adverse effects on the blood. Furthermore, biochemical blood indexes, including gamma‐glutamyl transferase (γ‐GT), aspartate aminotransferase (AST), blood urea nitrogen (BUN), creatine kinase (CK), and aminotransferase (ALT), were measured. The results did not indicate any obvious blood toxicity, suggesting that Fe‐SAC is safe for usage (Figure 6B–F). This finding was further confirmed by the discovery that every blood parameter tested during hematology analysis was within the usual range (Figure 6G). To assess the potential pathological changes, the main organs of the mice were subjected to H&E staining (Figure 6H). The outcomes revealed no side effects or pathological changes in the organs, indicating that Fe‐SAC did not cause any harm to the vital organs of the mice. Taken together, it could be concluded that Fe‐SAC exhibits negligible toxicity and is safe for use, ensuring its practical application as an antibacterial agent.

## 3 Conclusion

In this study, we presented a five‐coordinated structure Fe‐SAC with a high metal loading of 4.3 wt.% composed of nitrogen‐doped carbon. The maximized utilization of atoms and well‐defined active sites have greatly enhanced the catalytic performance of these nanozymes, surpassing that of conventional nanozymes. Fe‐SAC exhibits superior photothermal performance and increases the effectiveness of bactericidal POD‐mimic reactions for disrupting the compact biofilm and killing the bacteria. Fe‐SAC also exhibits NIR‐augmented POD‐like catalytic activity and GSH‐depleting capacity, which greatly suppresses the innate antioxidant defense of MRSA for photothermal‐boosted catalytic therapy. Thus, Fe‐SAC shows a remarkable multiscale antibacterial against MRSA infection, including biofilm penetration (extracellular level), bacterial capture (cellular level), and GSH depletion (intracellular level), which improves the therapeutic outcomes against MRSA infections both in vitro and in vivo. Moreover, the catalytic activity of Fe‐SAC may be accurately controlled by NIR laser irradiation, providing a potential method for the targeted treatment of infectious illnesses. Together, these findings indicated that the precise single‐atom nanozymes offer new insights into the catalytic mechanism and rational design, with significant potential to become the next‐generation nanozymes.

## 4 Experimental Section

### Reagents and Materials

FeCl_3_·6H_2_O, polyvinylpyrrolidone, and urea were bought from Macklin Reagent (Shanghai, China). 2‐Methylimidazole (2‐MI) was purchased from Bide Pharmatech_._ Ltd (Shanghai, China). Methanol and dimethyl sulfoxide (DMSO) were obtained from Sinopharm Chemical Reagent (Shanghai, China). 5, 5′‐dithiobis (2‐nitrobenzoic acid) (DTNB), 3,3′,5,5′‐tetramethyl‐benzidine (TMB), glutathione (GSH), and glacial acetic acid were purchased from Aladdin Bio‐Chem Technology Co (Shanghai). Agar powder and tryptone soya broth (TSB) were purchased from Solarbio (Beijing, China). Live/Dead staining kit and ROS staining test kit were commercially obtained from Beyotime Biotechnology (Shanghai, China). All chemical reagents were obtained from commercial companies and used as applicable without further purification.

### Synthesis of Fe‐SAC

The synthesis procedure of Fe‐SAC nanozyme was similar to the reported Co single‐atom catalyst.^[^ ^30^ ^]^ Typically, the mixture of FeCl_3_·6H_2_O (1.08 g), polyvinylpyrrolidone (PVP, 6.0 g) and urea (60 g) was reacted under 200 °C for 6 h. The resulting brown solid was heated at 900 °C at a rate of 5 °C min^−1^ for 120 min in an argon environment. Finally, the black product yield (3.75 g) was collected. The nitrogen‐doped carbon (NC) was synthesized via a process to that Fe‐SAC in the absence of FeCl_3_·6H_2_O.

### Characterization of Fe‐SAC

Transmission electron microscopy (TEM) images were used from a JEM 2100 Plus Microscope (JEOL Ltd., Tokyo, Japan) operating at 200 kV. The aberration‐corrected scanning transmission electron microscopy (AC‐STEM) images were collected by a JEM‐ARM200F transmission electron microscope, equipped with a probe spherical aberration corrector. The X‐ray diffraction (XRD) patterns were collected on a SmartLab X‐ray diffractometer with a scan rate of 2° min^−1^ over the 2*θ* range of 10–60°. X‐ray photoelectron spectroscopy (XPS) (ESCALAB, 250Xi, Thermo Fisher Scientific, USA) was used to examine the chemical states of the catalysts. A series of X‐ray absorption fine structure (XAFS) spectra was collected at Shanghai Synchrotron Radiation Facility's BL14W1 station. Fe foil and Fe_2_O_3_ were used as references and measured simultaneously. The ROS levels were measured by electron spin resonance (ESR) investigations using a Bruker EMX A300 spectrometer.

### Photothermal Performance and Photostability of Fe‐SAC

To evaluate the photothermal effect of Fe‐SAC, 1.0 mL dispersed aqueous solution of Fe‐SAC (0, 10, 25, 50, and 100 µg mL^−1^) was exposed to an 808 nm laser (1.0 W cm^−2^) for 10 min. Fe‐SAC dispersion suspension (50 µg mL^–1^) was exposed to an 808 nm laser for 10 min at three power densities (0.5, 1.0, and 2.0 W cm^−2^). An infrared thermal imaging camera (TIC) recorded the temperature in real time. Five laser cycles were employed to determine the photothermal stability of Fe‐SAC, while its photothermal conversion efficiency was computed as described previously.^[^ ^31^ ^]^

### Peroxidase‐Like Properties and Chemical Kinetics Analyses of Fe‐SAC

TMB probe was used as a signal to visualize and monitor the •OH generation. The POD‐like properties of Fe‐SAC were assessed by UV‐vis spectrometer to calculate the absorption of oxidized TMB (oxTMB) at 652 nm. Briefly, Fe‐SAC dispersion (10 µg mL^–1^) was mixed with aqueous solutions of TMB (0.5 mM, dissolved in DMSO) and H_2_O_2_ (0, 0.05, 0.1, 0.2, 0.5, and 1.0 mM) in 1 mL deionized water and incubated at room temperature (25 °C); the absorbance of oxTMB and color change were monitored. The pH‐dependent catalytic performance of Fe‐SAC was determined in disodium hydrogen phosphate with citric acid. ESR spectroscopy was carried out on Bruker EMX PLUS(USA). 5, 5‐ Dimethyl‐1‐pyrroline N‐oxide (DMPO) was used as the spin trap to ensure the catalytic activity and free radicals. The chemical kinetics of Fe‐SAC (50 µg mL^–1^) were assessed in the presence of varying concentrations of H_2_O_2_ (1, 6, 8, 12, 16, and 32 mm), and the suspension's absorbance was measured at 652 nm. After obtaining the Michaelis‐Menten curve, the Vmax and Km were computed as described for the Lineweaver‐Burk plot.^[^ ^32^ ^]^

### GSH‐Depleted Characteristics of Fe‐SAC

The decrease in GSH level was confirmed based on the absorbance measurement of the DTNB probe at 420 nm. Specifically, 100 µL Fe‐SAC was combined with varying amounts of GSH (0.1 M) in PBS at room temperature. Following incremental additions of 100 µL in PBS (pH 7.4), the mixture was exposed to 0.1 mM DTNB for 3 min and the absorbance was measured at 412 nm. GSH oxidation was calculated according to the following equation.^[^ ^33^ ^]^

(1) $$\mathrm{Loss}\;\mathrm{of}\;\mathrm{GSH}\left(\%\right)=\frac{\mathrm{ODcontrol}-\mathrm{ODsample}}{\mathrm{ODcontrol}}\times 100\%$$

### Antimicrobial Effect of Fe‐SAC In Vitro

Gram (+) bacterial cell strain: MRSA (Mu50) and Gram (‐) bacterial: *E. coli* (DH5α) was utilized as the model organisms. At OD = 0.5 (600 nm), the bacterial suspension was divided into eight groups: (1) bacteria; (2) bacteria + H_2_O_2_; (3) bacteria + Fe‐SAC; (4) bacteria + Fe‐SAC + H_2_O_2_; (5) bacteria + NIR‐I light; (6) bacteria + NIR‐I light + H_2_O_2_; (7) bacteria + Fe‐SAC + NIR‐I light; (8) bacteria + Fe‐SAC + H_2_O_2_+ NIR‐I light. All groups were suspended in sterile PBS. The spread‐plated method was used to evaluate the antibacterial effect of nanoparticles. Briefly, 1 × 10^8^ CFU mL^−1^ bacterial suspension, 0.1 mM H_2_O_2_, and Fe‐SAC (50 µg mL^–1^) were mixed in 1 mL PBS. Groups (5)–(8) were further exposed to NIR‐I (808 nm, 1.0 W cm^−2^) for 5 min and every group was grown at 37 °C for 2 h. The agar plate was coated with a 25 µL diluted bacterial culture and, incubated at 37 °C for 16 h. Finally, the bacterial colonies were calculated by the plated counting method.

### Live/Dead Staining

SYTO‐9/PI staining was utilized to evaluate the bacteria's capacity to survive various treatments. Propidium iodide (PI, a red fluorescent dye) and SYTO‐9 (a green fluorescent dye) were applied to co‐stain all groups for about 20 min, followed by three PBS washes. A confocal laser scanning microscope was used to monitor the live (green fluorescence) and dead (green fluorescence) cells.

### Disruption of Bacterial Biofilm Assay

To prepare planktonic bacteria, 20 µL of 1.0 × 10^8^ CFU mL^−1^ of MRSA was dispensed in 1.0 mL TSB medium was filled with a 24‐well plate and cultured overnight at 37 °C to establish an intact biofilm. Similarly, eight groups; 1) bacteria; 2) bacteria + H_2_O_2_; 3) bacteria + Fe‐SAC; 4) bacteria + Fe‐SAC + H_2_O_2_; 5) bacteria + NIR‐I light; 6) bacteria + NIR‐I light + H_2_O_2_; 7) bacteria + Fe‐SAC + NIR‐I light; 8) bacteria + Fe‐SAC + H_2_O_2_+ NIR‐I light, were employed to assess the disruption of biofilm. Then, the supernatant from each well was collected and dried to fix the MRSA biofilm, which was subsequently stained with 0.5% crystal violet dye for 15 min, rinsed twice with PBS, and dried at room temperature for 1 h. Finally, the stained biofilms were dissolved in glacial acetic acid and absorbance at 540 nm was measured. The integrity of the MRSA biofilm was verified using the SYTO‐9/PI live/dead staining kit.

### Cell Culture and Cytotoxicity of Fe‐SAC In Vitro

L929 cell lines were maintained in a humidified atmosphere containing 5% CO_2_ at 37 °C and cultured in high‐glucose Dulbecco's modified Eagle medium (DMEM; Gibco, USA) supplemented with 10% fetal bovine serum (FBS; Gibco, USA), 100 U mL^−1^ penicillin, and 100 µg mL^−1^ streptomycin (Gibco, USA). CCK‐8 assay was performed to determine the cytotoxicity of Fe‐SAC. Briefly, 0.5 × × 10^4^ cells per well were grown in a 96‐well plate for 24 h. At 60–70% confluency, the medium was replaced by Fe‐SAC DMEM dispersion (0, 5, 10, 20, 40, 60, 80, 100, 200 µg mL^−1^) for 24 h. After the addition of CCK‐8 reagent (v/v, puro DMEM: CCK‐8 reagent = 9:1), the reaction was incubated at 37 °C for an additional 2 h before measuring at 450 nm.

### Antimicrobial Performance of Fe‐SAC In Vivo

The approval for animal experiments was obtained from The Experimental Animal Ethics Review Committee of Wenzhou Medical University. Male balb/c mice weighing <20 g at 7–8 weeks of age had a subcutaneous MRSA infection in the back. After a day, all mice were randomly divided into five groups (n = 6 per group): (1) PBS+NIR light; (2) Fe‐SAC; (3) Fe‐SAC + NIR light; (4) Fe‐SAC + H_2_O_2_; (5) Fe‐SAC + H_2_O_2_+ NIR light. For groups (2)–(5), Fe‐SAC suspension (100 µL, 50 µg mL^−1^) was administered into the abscess location. Groups (1) and (3)‐(5) were exposed to an 808 nm laser (1.0 W cm^−2^) for 5 min. A thermal imaging camera was used to track real‐time temperature. Simultaneously, pictures of the abscess location were taken every 2 days. All mice were executed after receiving treatment for 10 days, the wound sites were gathered, and the main organs were excised. The infected skin was further evaluated for inflammatory reaction and wound healing by H&E staining, Masson staining, and immunofluorescence. Additionally, the vital organs, such as the liver, spleen, kidney, heart, and lung, were harvested and evaluated using H&E staining

### Statistical Analysis

At least three replications of each experiment were conducted. Data was presented as the mean ± standard deviation and analyzed using GraphPad Prism software (version 9.0). the statistically significant values were estimated using two‐sided One‐way analysis of variance (ANOVA) tests and student's t‐tests with a statistical significance threshold of ^*^p < 0.05, ^**^p < 0.01, and ^***^p < 0.001.

## Conflict of Interest

The authors declare no conflict of interest.

## Author Contributions

Q.L. and X.L. contributed equally to this work. Q.L. and X.L.L. conceived of and designed the experiments. X.L.L. and X.J.H. conducted material synthesis and the characterizations of the materials. Q.L., Z.C., and D.Y.W. performed the catalytic test and photothermal experiments. Q.L., X.L.L, X.J.H., Z.C., and D.Y.W. contributed to data analysis. Q.L. and X.L.L. wrote the manuscript. J.L.S. and L.J. supervised the project and revised the manuscript. Each author reviewed the findings, provided feedback on the text, and approved the edited version of this work.

## Supporting information

Supporting Information

## Data Availability

The data that support the findings of this study are available in the supplementary material of this article.
