# Correction to “Iron Single‐Atom Nanozyme With NIR Enhanced Catalytic Activities for Facilitating *MRSA*‐Infected Wound Therapy”

**DOI:** 10.1002/advs.74838

**Published:** 2026-03-13

Qian Liu, Xueliang Liu, Xiaojun He, Danyan Wang, Chen Zheng, Lin Jin, Jianliang Shen

https://doi.org/10.1002/advs.202308684. Adv Sci (Weinh) 2024 April; 11(15):2308684.

The published version of our manuscript has an error in one of the figures included in the paper. On page 13 of the published paper, an error was identified in Figure 6, where the kidney images for the Fe‐SAC group and the Fe‐SAC+NIR‐I group were mistakenly swapped. As a result, the figure does not accurately reflect the experimental results. While the incorrect images do not align with the original data, this error does not affect the overall conclusions of the study.

To address this issue, we have re‐examined the original data and prepared the correct images to replace the erroneous ones. The corrected image is shown below:


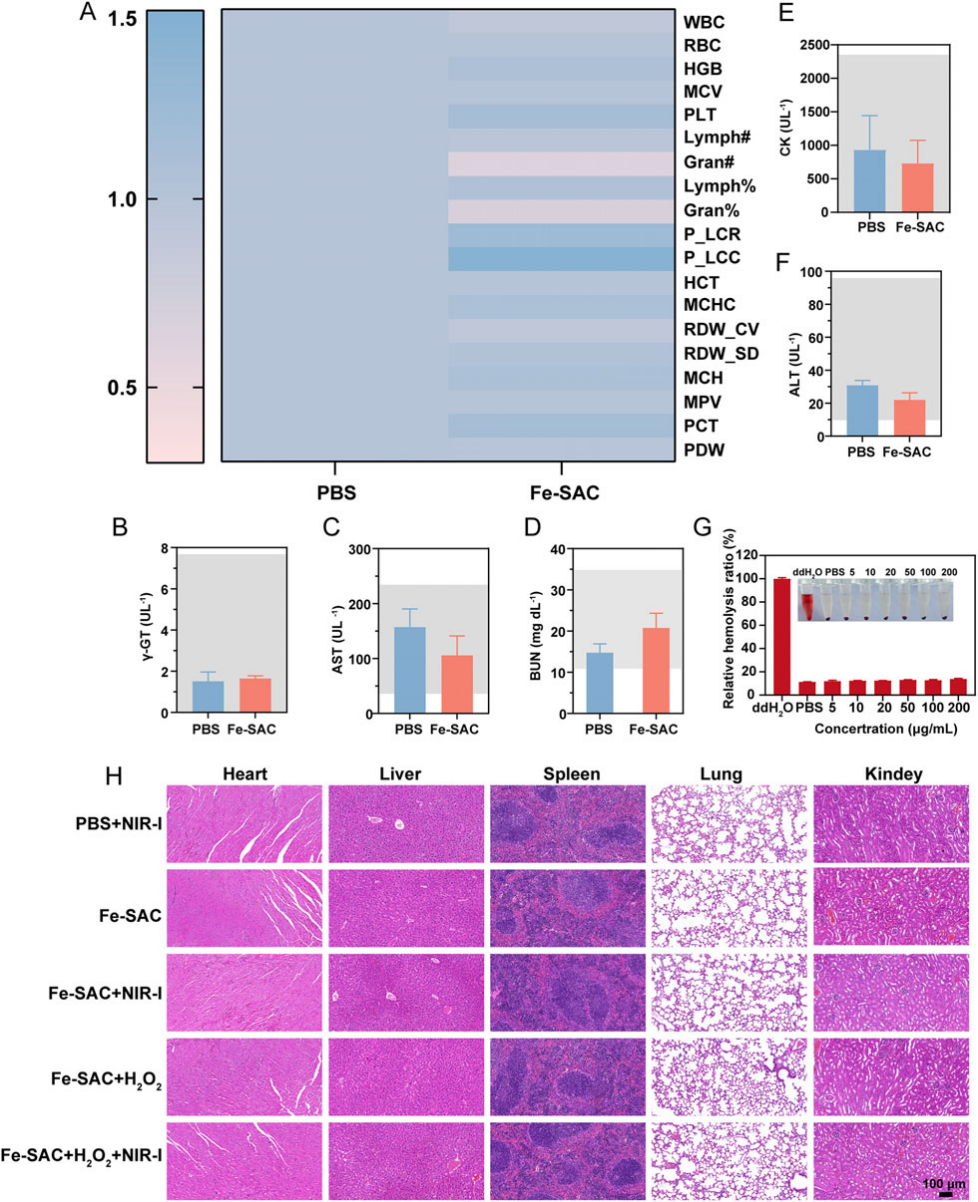


We sincerely apologize for this oversight and any inconvenience it may cause to the editorial team and the readers.

