# Supplementary material for: Iron‐Single‐Atom Nanozyme with NIR Enhanced Catalytic Activities for Facilitating MRSA‐Infected Wound Therapy
Source: Adv Sci (Weinh). 2024 Feb 8;11(15):2308684. doi: 10.1002/advs.202308684 (PMC11022696; doi:10.1002/advs.202308684)
Supplement: Supplementary file 1 — Supporting Information [file ADVS-11-2308684-s001.pdf]

ADVANCED SCIENCE
Open Access

Supporting Information

for *Adv. Sci.*, DOI 10.1002/advs.202308684

Iron-Single-Atom Nanozyme with NIR Enhanced Catalytic Activities for Facilitating MRSA-Infected Wound Therapy

Qian Liu, Xueliang Liu, Xiaojun He, Danyan Wang, Chen Zheng, Lin Jin and Jianliang Shen**

Supporting Information

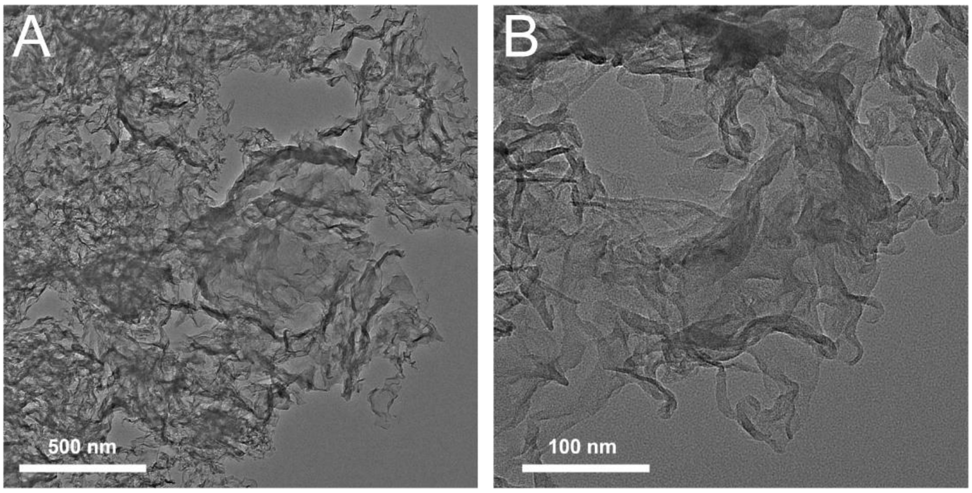

Figure S1. (A) TEM images of NP with a scale bar of 500 nm and (B) enlarged TEM image of (A) with a scale bar of 100 nm.

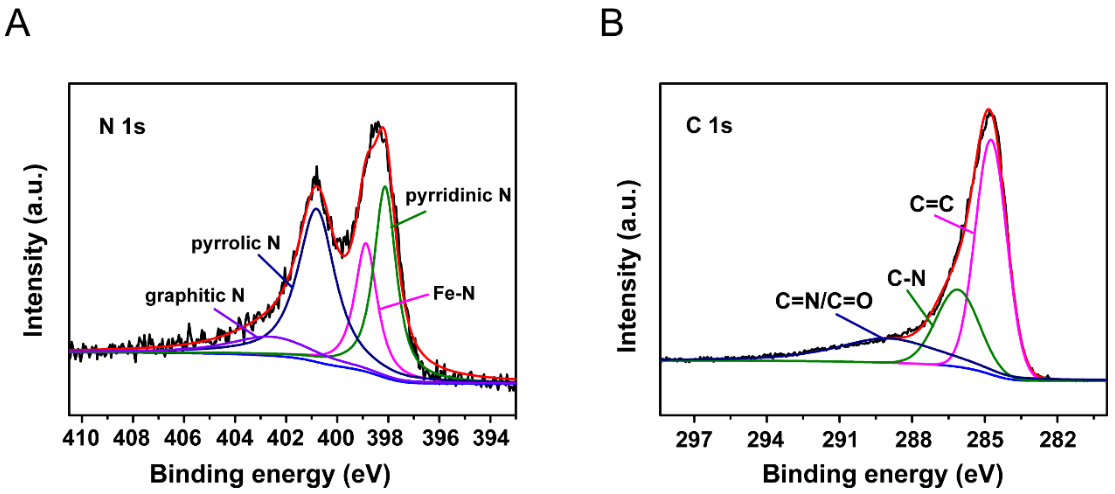

Figure S2. High-resolution XPS spectra of N 1s (A) and C 1s (B) of Fe-SAC.

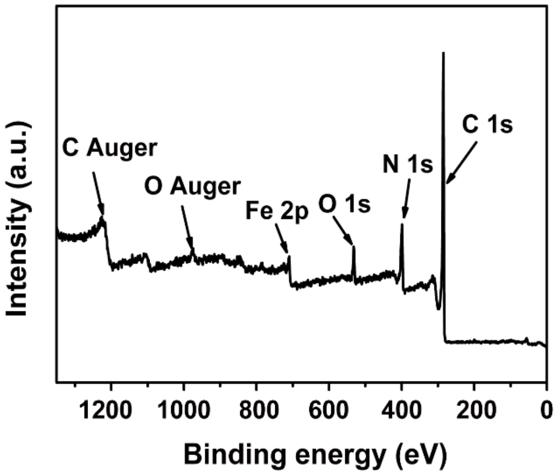

Figure S3. The XPS survey spectrum of Fe-SAC.

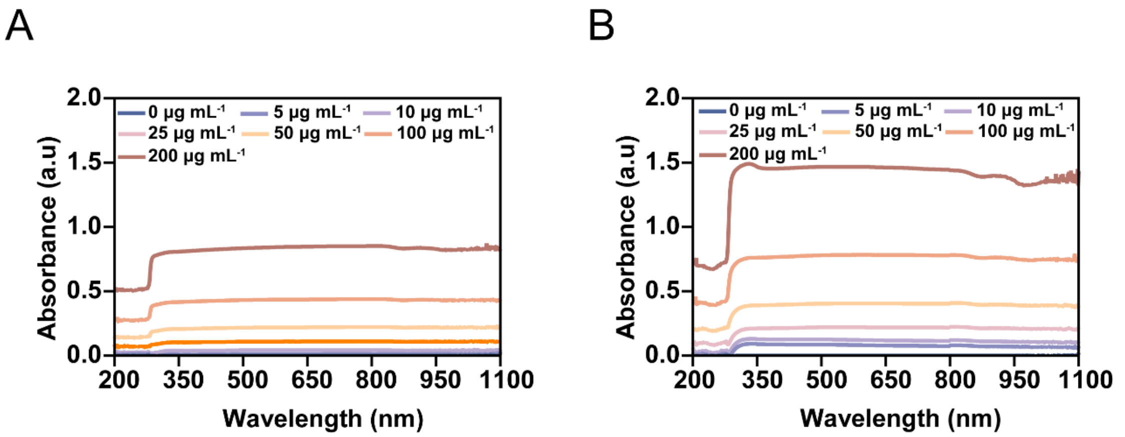

Figure S4. UV absorption spectrum of NP (A) and Fe-SAC (B) with different concentrations.

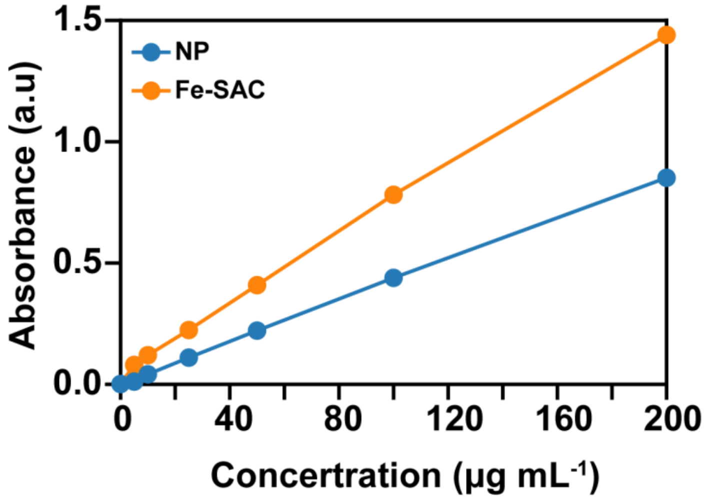

Figure S5. Comparison of UV absorption between NP and Fe-SAC at 808 nm.

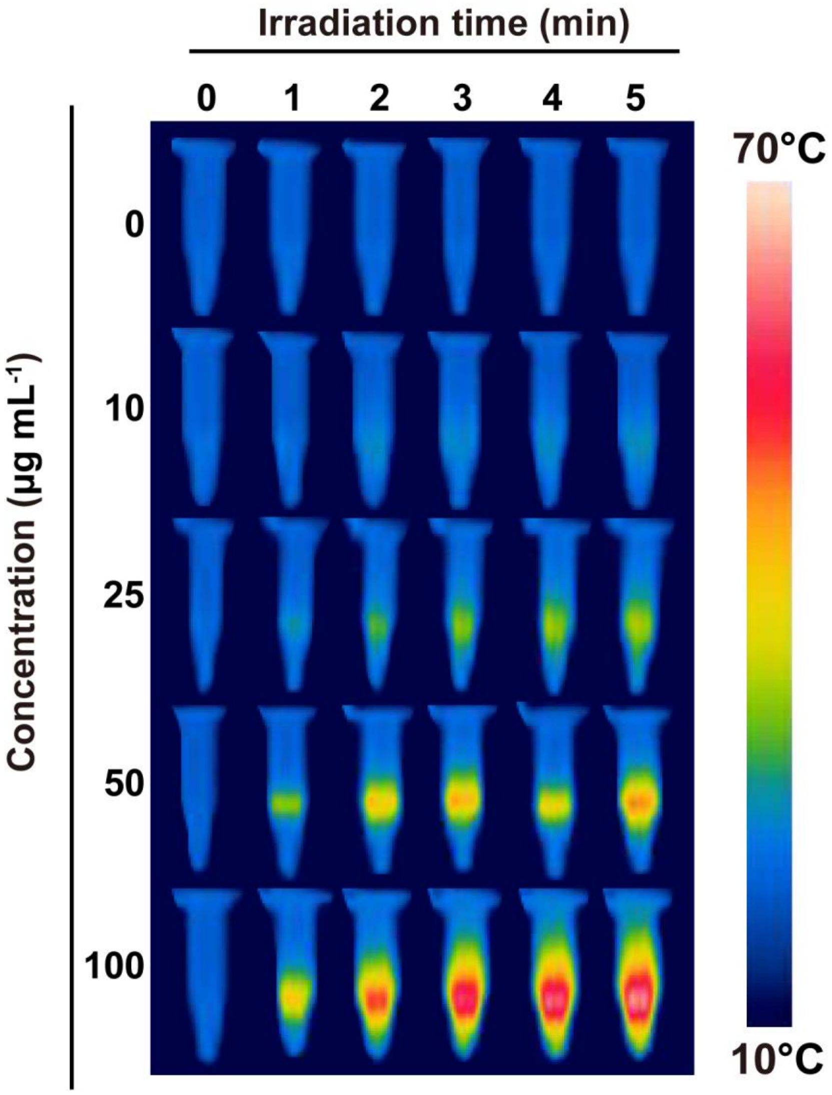

Figure S6. Infrared thermal images of various concentrations of Fe-SAC upon an 808 nm laser irradiation.

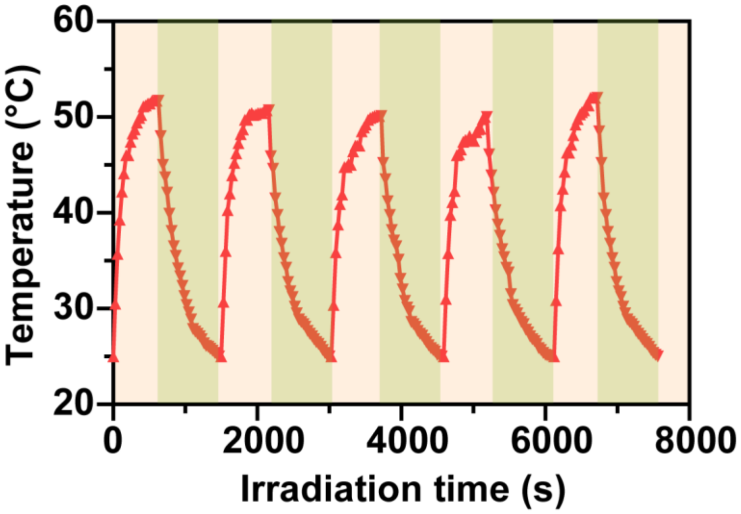

Figure S7. Photothermal stability during five cycles of on/off NIR-I irradiation.

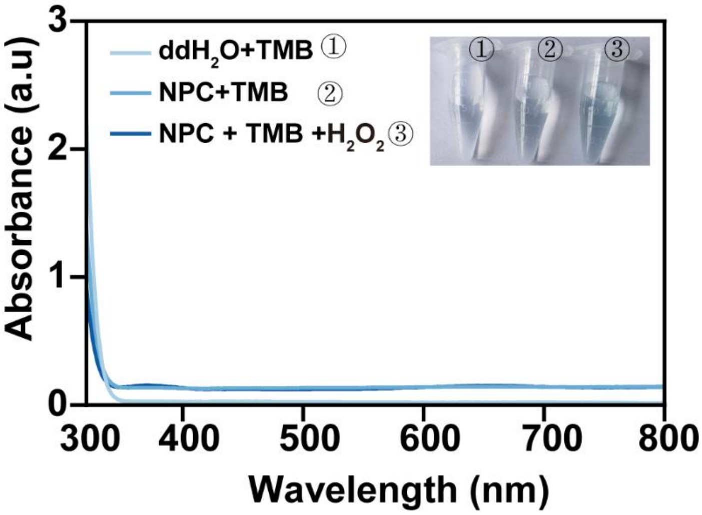

Figure S8. TMB absorption with NC in ddH_2O and Relevant photographs of TMB color change.

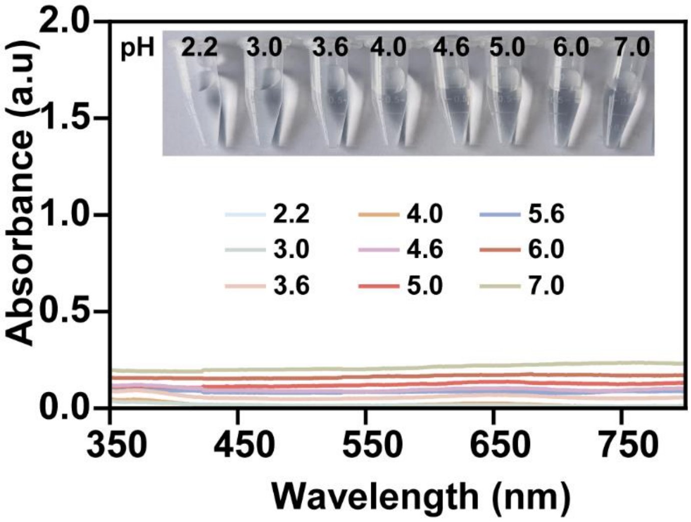

Figure S9. TMB absorption at 652 nm with dispersed NC suspension under different pH phosphate buffers and Relevant photographs of TMB color change.

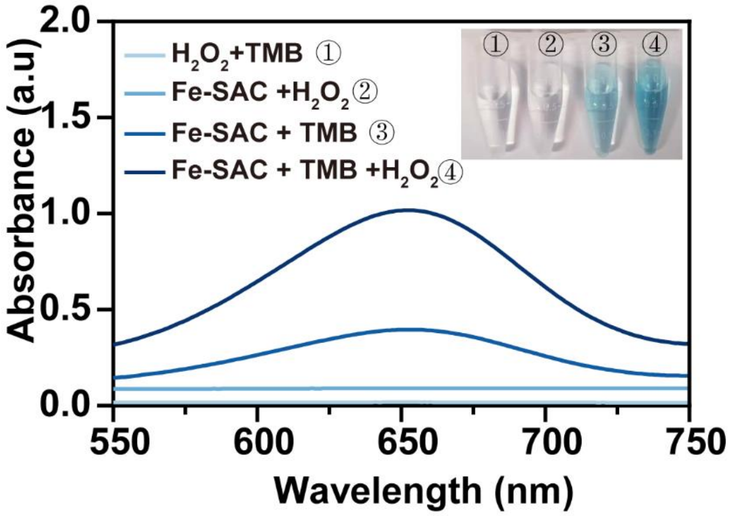

Figure S10. TMB absorption with Fe-SAC nanoparticles in ddH_2O and Relevant photographs of TMB color change.

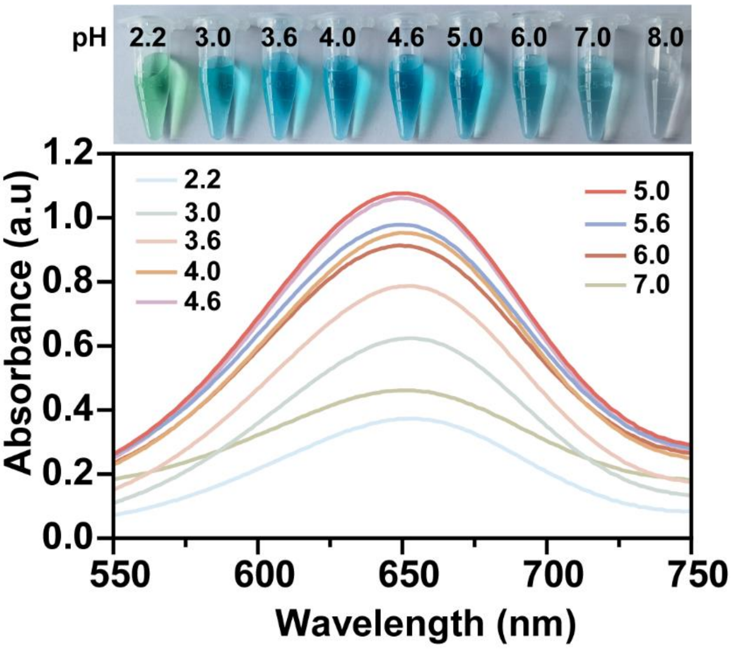

Figure S11. TMB absorption at 652 nm with dispersed Fe-SAC suspension under different pH phosphate buffers and Relevant photographs of TMB color change.

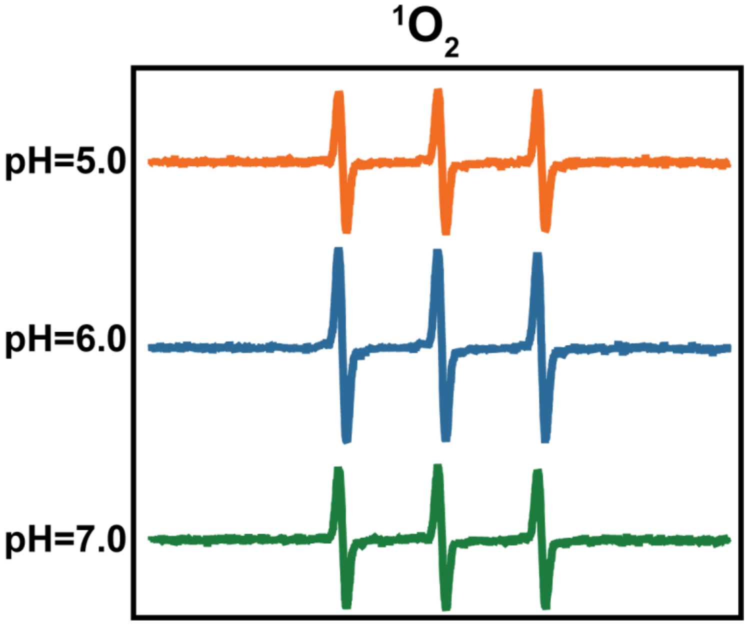

Figure S12. ESR spectrum of singlet oxygen trapped by DMPO under different pH conditions.

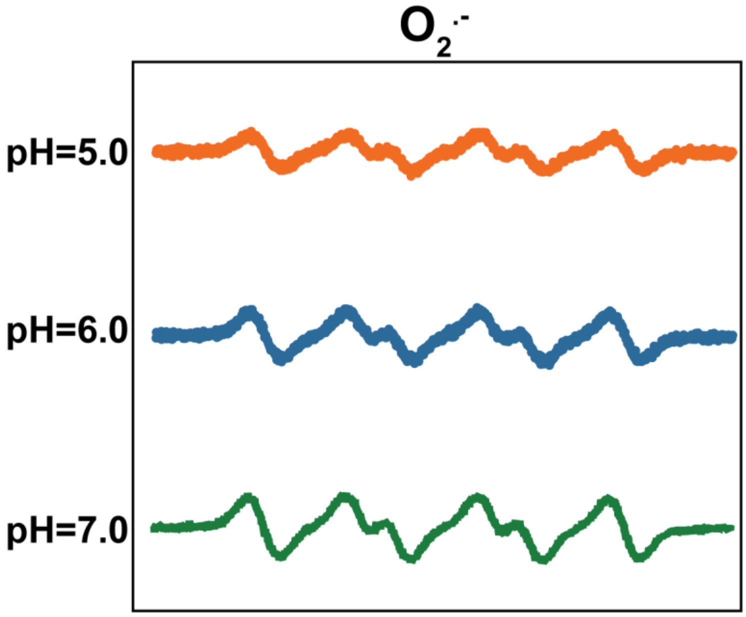

Figure S13. ESR spectrum of superoxide anion by DMPO under different pH conditions.

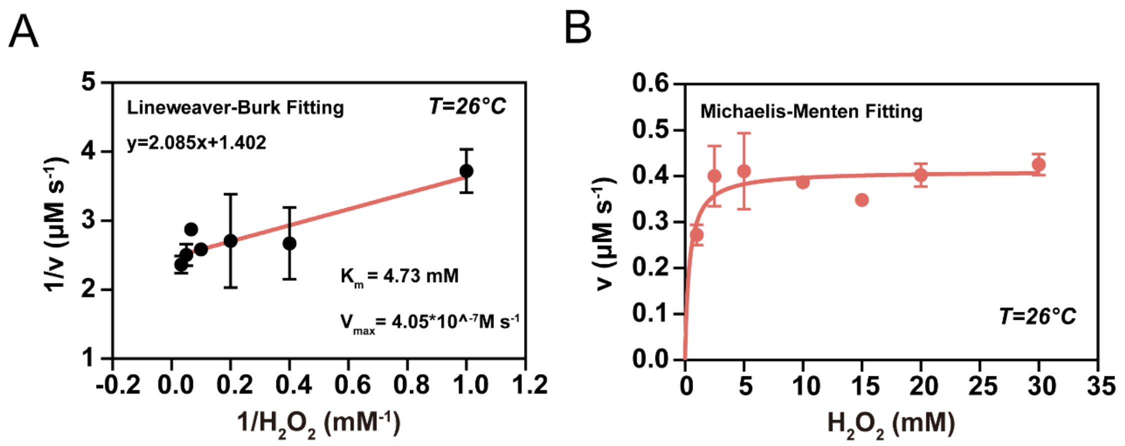

Figure S14. (A) Lineweaver-Burk plot for Fe-SAC with H_2O_2 as a substrate and (B) Michaelis-Menten kinetic analysis of Fe-SAC with H_2O_2 as a substrate at room temperature. Data of each of the three independent experiments (n = 3) are presented as mean ± standard deviation (SD). The following criteria were used to assess statistical significance: *$P < 0.05$, **$P < 0.01$, ***$P < 0.001$.

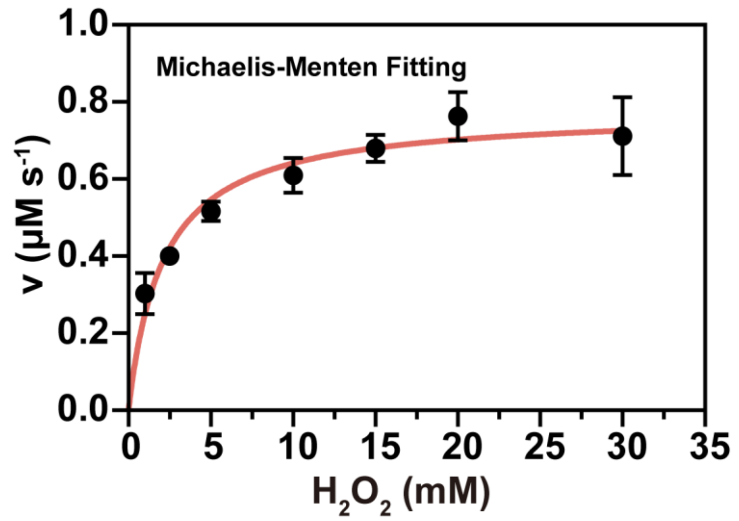

Figure S15. Michaelis-Menten kinetic analysis of Fe-SAC with H_2O_2 as a substrate. Data of each of the three independent experiments (n = 3) are presented as mean ± standard deviation (SD). The following criteria were used to assess statistical significance: *$P < 0.05$, **$P < 0.01$, ***$P < 0.001$.

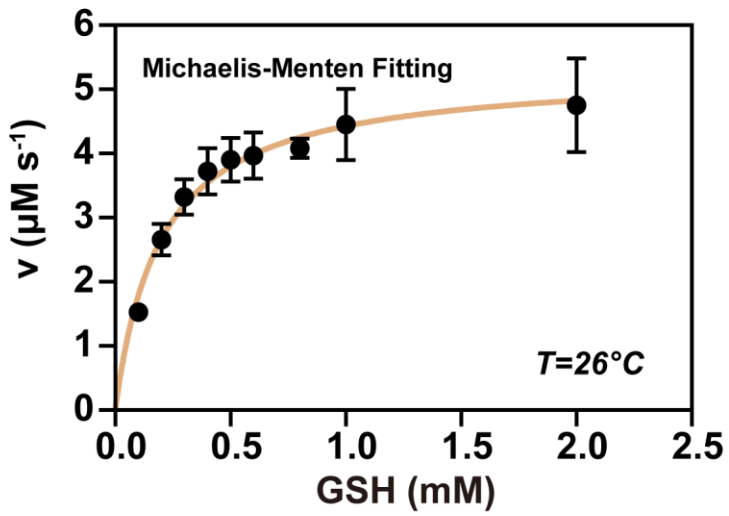

Figure S16. Michaelis-Menten kinetic analysis of Fe-SAC with GSH as a substrate. Data of each of the three independent experiments (n = 3) are presented as mean ± standard deviation (SD). The following criteria were used to assess statistical significance: $*P < 0.05$, $**P < 0.01$, $***P < 0.001$.

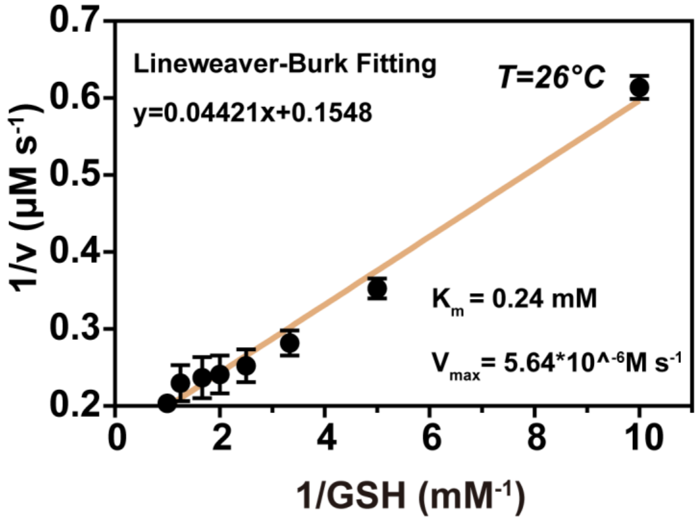

Figure S17. Lineweaver-Burk plot for Fe-SAC with GSH as a substrate. Data of each of the three independent experiments (n = 3) are presented as mean ± standard deviation (SD). The following criteria were used to assess statistical significance: *$P < 0.05$, **$P < 0.01$, ***$P < 0.001$.

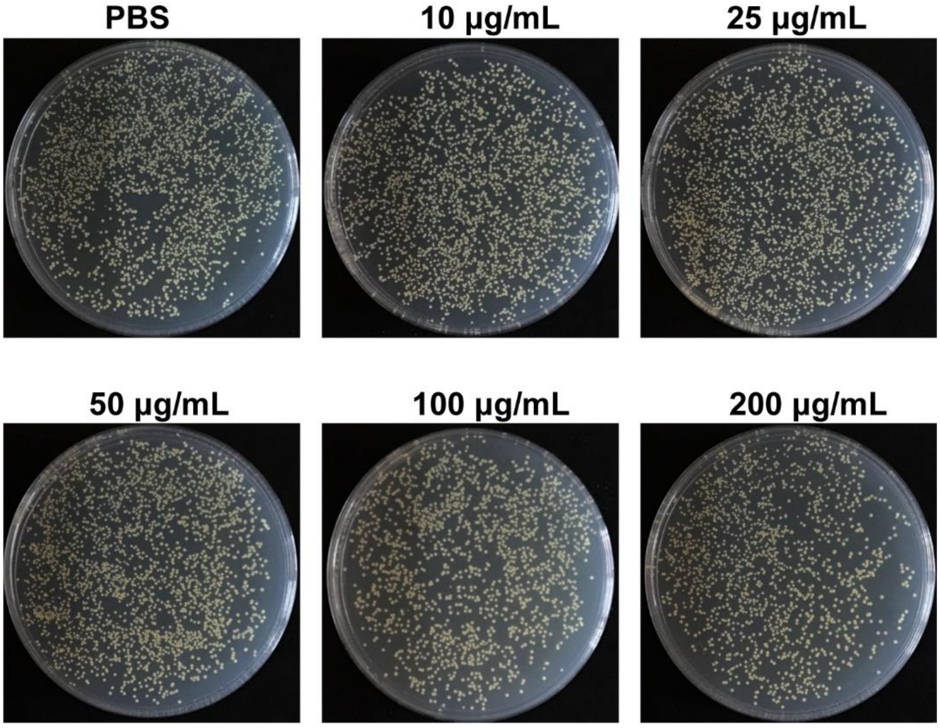

Figure S18. In *vitro* antibacterial effect of Fe-SAC (0, 10, 25, 50, 100, 200 μg/mL). Data of each of the three independent experiments (n = 3) are presented.

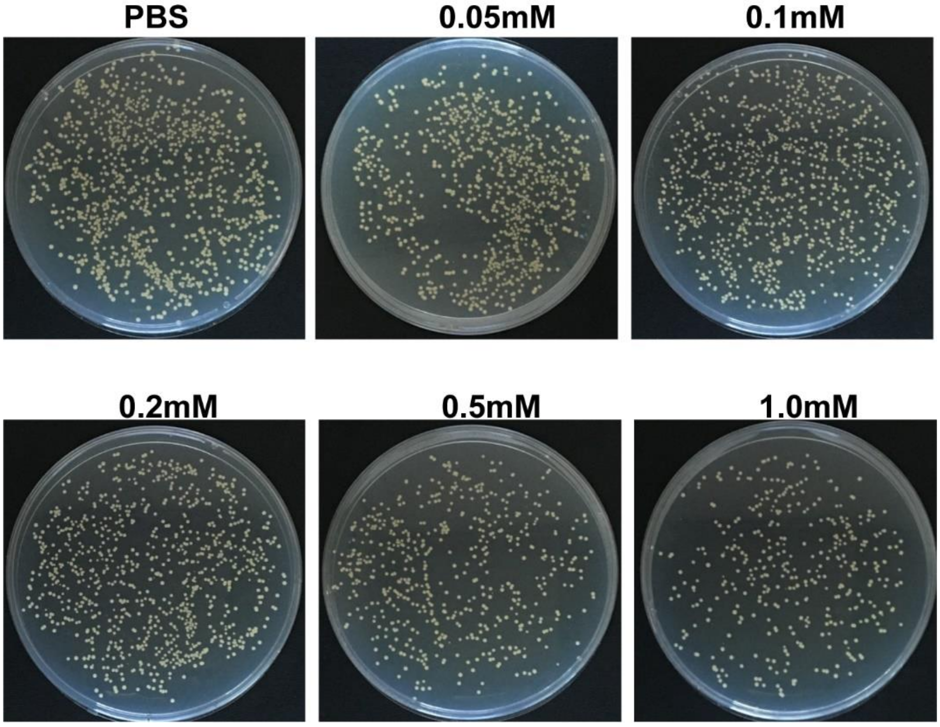

Figure S19. In *vitro* antibacterial effect of H_2O_2 (0, 0.05, 0.1, 0.2, 0.5, 1.0 mM). Data of each of the three independent experiments (n = 3) are presented.

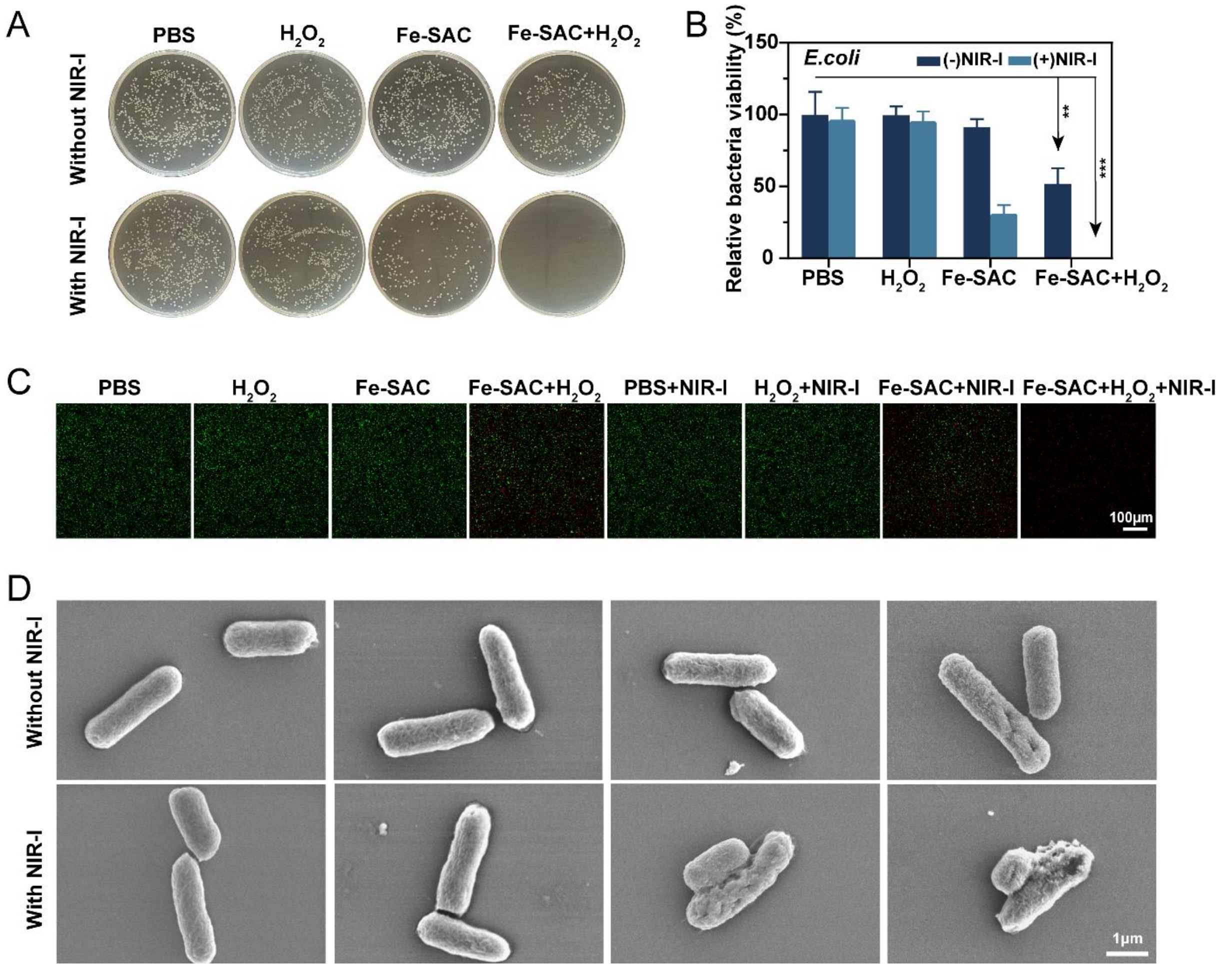

Figure S20. Antibacterial performance of Fe-SAC *in vitro*. (A) Agar plate photographs of *E.coli* bacterial colonies by Fe-SAC under NIR-I irradiation, PBS as control. (B) Relative bacterial viability of *E.coli*. (C) Live/dead staining of *MRSA*. (D) SEM images of *E.coli* after different treatments. Data of each of the three independent experiments (n = 3) are presented as mean ± standard deviation (SD). The following criteria were used to assess statistical significance: $*P < 0.05$, $**P < 0.01$, $***P < 0.001$.

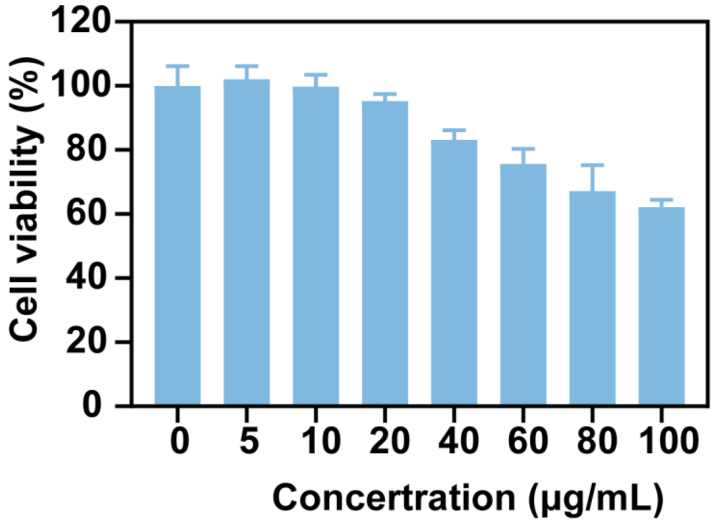

Figure S21. Relative cell viabilities of L929 cells after incubation with different concentrations of Fe-SAC (0, 5, 10, 20, 40, 60, 80, and 100 μg/mL) for 24 h. Data of each of the three independent experiments (n = 3) are presented as mean ± standard deviation (SD). The following criteria were used to assess statistical significance: *$P < 0.05$, **$P < 0.01$, ***$P < 0.001$.

Table S1. Elemental composition of different samples measured by XPS.

Samples	C	N	O	Fe
Fe-SAC	73.3%	18.5%	3.9%	4.3%

Table S2. The catalytic activity for different single atom nanozymes.

Samples	Nanozyme type	Substrate	K_m [mmol L^{-1}]	V_{max} [μmol L^{-1} s^{-1}]	Loading	reference
Fe–N–C SAN	POD	H_2O_2	28.3	0.4285	1.85 at%	[1]
FeN_5 SA/CNF	OXD	TMB	0.148	0.758	1.2 wt%	[2]
SAF NCs	POD	H_2O_2	11.95	0.223	1.36 wt%	[3]
Fe-N_5 SAzyme	POD	H_2O_2	11.2	2.96	0.29 at%	[4]
FePN SAzyme	POD	H_2O_2	2.61	3.44	/	[5]
PSAF NCs	POD	H_2O_2	/	/	1.54 wt%	[6]
Fe-N-C	POD	H_2O_2	4.84	0.118	/	[7]
Fe–N–C	POD	H_2O_2	12.2	0.356	1.3 wt%	[8]
Fe–N-rGO	POD	H_2O_2	43	1.44	1.8 wt%	[9]
FeSAs	POD	H_2O_2	5.87	0.617	0.75 wt%	[10]
Fe-SAC	POD	H_2O_2	1.49	0.713	4.3 wt%	this work

Reference

[1] X. Niu, Q. Shi, W. Zhu, D. Liu, H. Tian, S. Fu, N. Cheng, S. Li, J. N. Smith, D. Du, Y. Lin, *Biosens Bioelectron* **2019**, *142*, 111495, https://doi.org/10.1016/j.bios.2019.111495.
[2] L. Huang, J. Chen, L. Gan, J. Wang, S. Dong, Single-atom nanozymes. *Science Advances*. 5, eaav5490 **2019**, https://doi.org/10.1126/sciadv.aav5490.
[3] M. Huo, L. Wang, H. Zhang, L. Zhang, Y. Chen, J. Shi, *Small* **2019**, *15* (31), e1901834, https://doi.org/10.1002/smll.201901834.
[4] B. Xu, S. Li, L. Zheng, Y. Liu, A. Han, J. Zhang, Z. Huang, H. Xie, K. Fan, L. Gao, H. Liu, *Advanced Materials* **2022**, *34* (15), https://doi.org/10.1002/adma.202107088.
[5] Q. Xu, Y. Hua, Y. Zhang, M. Lv, H. Wang, Y. Pi, J. Xie, C. Wang, Y. Yong, *Advanced Healthcare Materials* **2021**, *10* (22), https://doi.org/10.1002/adhm.202101374.
[6] M. Huo, L. Wang, Y. Wang, Y. Chen, J. Shi, *ACS Nano* **2019**, https://doi.org/10.1021/acsnano.9b00457.
[7] Y. Feng, J. Qin, Y. Zhou, Q. Yue, J. Wei, *Journal of Colloid and Interface Science* **2022**, *606*, 826, https://doi.org/10.1016/j.jcis.2021.08.054.
[8] L. Jiao, W. Xu, H. Yan, Y. Wu, C. Liu, D. Du, Y. Lin, C. Zhu, *Anal Chem* **2019**, *91* (18), 11994, https://doi.org/10.1021/acs.analchem.9b02901.
[9] M. S. Kim, J. Lee, H. S. Kim, A. Cho, K. H. Shim, T. N. Le, S. S. A. An, J. W. Han, M. I. Kim, J. Lee, *Advanced Functional Materials* **2019**, *30* (1), https://doi.org/10.1002/adfm.201905410.
[10] H. Liu, X. Liu, H. Wang, J. Ren, X. Qu, *Small* **2023**, *19* (37), e2207510, https://doi.org/10.1002/smll.202207510.
